# Integrating network pharmacology, microbiomics, and metabolomics to uncover the therapeutic effect of Liubao tea on osteoarthritis

**DOI:** 10.3389/fimmu.2026.1746350

**Published:** 2026-02-19

**Authors:** Guoping Le, Riyou Wen, Zhifa Huang, Huaixi Fang, Jianwei Zheng, Yong Wang, Hanwen Luo

**Affiliations:** Department of Joint Osteopathy, Liuzhou Worker’s Hospital, The Fourth Affiliated Hospital of Guangxi Medical University, Liuzhou, Guangxi, China

**Keywords:** gut microbiota, Liubao tea, network pharmacology, osteoarthritis, pyrimidine metabolism

## Abstract

**Background:**

Osteoarthritis (OA) is a debilitating joint disorder for which with no effective disease-modifying drugs are currently available. Liubao tea, a traditional Chinese post-fermented tea, exhibits diverse bioactivities, including anti-inflammatory properties and the ability to regulate gut microbiota. However, its potential therapeutic efficacy and underlying mechanism in the context of OA remain insufficiently elucidated.

**Methods:**

A mouse model of osteoarthritis (OA) induced by destabilization of the medial meniscus (DMM) was established, and the mice were treated with low- and high-dose Liubao tea extract. Micro-CT, histological staining (H&E, Safranin O-Fast Green), and enzyme-linked immunosorbent assay (ELISA) were performed to evaluate joint structure, cartilage damage, and inflammatory cytokine levels. 16S rRNA sequencing, fecal microbiota transplantation (FMT), and untargeted serum metabolomics were conducted to explore gut microbiota and metabolic changes. Additionally, Brequinar, a *de novo* pyrimidine synthesis inhibitor, was used to verify the role of pyrimidine metabolism. Ultra-performance liquid chromatography-tandem mass spectrometry (UPLC-MS/MS) was used to analyze the chemical components of Liubao tea. Network pharmacology was employed to identify the active components and their potential targets in OA treatment. Molecular docking was performed to evaluate the interactions between key components and hub targets.

**Results:**

Liubao tea treatment significantly ameliorated DMM-induced OA progression, as evidenced by improved subchondral bone microarchitecture (increased bone volume/total volume [BV/TV], trabecular number [Tb.N], trabecular thickness [Tb.Th]; decreased trabecular separation [Tb.Sp]), the reduced cartilage erosion (lowered the modified Mankin and OARSI scores), and the suppressed systemic inflammation (decreased interleukin [IL]-6, IL-1β, tumor necrosis factor [TNF]-α levels). Liubao tea remodeled gut microbiota homeostasis (increased α-diversity and altered bacterial taxa), and fecal microbiota transplantation (FMT) from Liubao tea-treated mice recapitulated its anti-OA effects. Metabolomic analysis revealed that Liubao tea significantly downregulated the pyrimidine metabolism pathway, and Brequinar treatment mimicked its therapeutic benefits, confirming the role of pyrimidine metabolism suppression in OA alleviation. UPLC-MS/MS and network pharmacology analyses identified 1,989 metabolites in Liubao tea, including 273 bioactive components (e.g., flavonoids, lignans) that targeted 324 OA-related genes. The molecular docking results demonstrated that Eupatilin, 5,6,7,8-Tetramethoxyflavone, and 5-Hydroxy-6,7,3’,4’,5’-Pentamethoxyflavone exhibited potential interactions with the hub targets TP53, IL6, and TNF.

**Conclusion:**

Liubao tea attenuates OA progression by modulating the composition of the gut microbiota and inhibiting the pyrimidine metabolism pathway, highlighting its potential as a novel natural therapeutic agent for OA.

## 1 Background

Osteoarthritis (OA) is one of the most common chronic joint diseases globally, particularly prevalent among the elderly (1). Its primary clinical features include joint pain, stiffness, and functional impairment, significantly impacting patients’ quality of life (1). However, despite extensive research on the pathophysiology of osteoarthritis over the past few decades, its exact mechanisms remain incompletely understood (2, 3). Currently, treatment for OA primarily focuses on symptom management, with a lack of drugs that can effectively modify disease progression (4). In Western medicine, commonly used drugs include non-steroidal anti-inflammatory drugs (NSAIDs), acetaminophen, and topical medications (4). However, these drugs typically provide only limited pain relief and come with various side effects. Therefore, researchers are exploring new treatment strategies, including the development of disease-modifying osteoarthritis drugs (DMOADs)”——DMOADs that target the pathophysiological processes of OA (5). At the same time, traditional Chinese medicine (TCM) has demonstrated unique advantages in the treatment of OA (6). With its holistic regulation and personalized therapy, TCM plays a significant role in alleviating symptoms, reducing adverse reactions, and improving patients’ quality of life (7, 8). For example, compound formulas and single herbs have shown remarkable effects in improving symptoms in patients with knee osteoarthritis (9). Additionally, TCM influences the pathogenesis of OA through mechanisms such as regulating gut microbiota and improving metabolic immunity (10, 11). Hence, exploration of the role of traditional Chinese medicine in osteoarthritis may provide more effective treatment options for patients with this disease.

Liubao tea is a traditional Chinese dark tea renowned for its unique flavor and potential health benefits (12). In recent years, the potential of Liubao tea in treating various diseases has attracted widespread attention. Studies have shown that Liubao tea possesses multiple biological activities, including antioxidant, anti-inflammatory, anti-obesity, and gut microbiota-regulating effects (13–16). For instance, Liubao tea extract has been shown to suppress obesity-related hyperlipidemia by regulating the AMPK/p38/NF-κB signaling pathway and modulating intestinal microbiota composition (15). Polyphenols isolated from Liubao insect tea alleviate dextran sulfate sodium (DSS)-induced experimental colitis through protecting intestinal barrier function and regulating gut microbiota balance (16). Additionally, Liubao tea extract ameliorates ovalbumin-induced allergic asthma in mice by reshaping the gut microbiota profile (17). In metabolic disease models, Liubao tea extract attenuates high-fat diet- and streptozotocin-induced type 2 diabetes by remodeling hepatic metabolic pathways and gut microbial communities (18). Furthermore, polyphenolic components of Liubao tea prevent carbon tetrachloride (CCl_4_)-induced hepatic injury in mice via their potent antioxidant capacities (13). Despite the well-documented therapeutic effects of Liubao tea in multiple disease models, its potential role and underlying mechanisms in OA remain unexplored. Hence, it is imperative to further investigate whether Liubao tea can ameliorate OA progression, which may provide novel insights into the development of natural product-based therapeutic strategies for this debilitating joint disorder.

Previous studies have demonstrated that integrating network pharmacology, fecal microbiota analysis, and metabolomics can effectively explore the therapeutic effects and underlying mechanisms of natural compounds (19). Therefore, in the present study, we employed network pharmacology to identify the potential targets of Liubao tea in the treatment of osteoarthritis, while investigating the impacts of Liubao tea treatment on the microbial composition and serum metabolomics of osteoarthritic mice via fecal microbiota and metabolomics analyses. This study could provide insights into the role of Liubao tea in osteoarthritis treatment.

## 2 Materials and methods

### 2.1 Drugs and reagents

Liubao tea (LPT) was purchased from China Tea Co., Ltd. (Wuzhou, Guangxi, China; Batch No.: S002/2022) and complied with the specifications of GB/T 32719.4-2016 (Brick-type Liubao Tea, Grade 2). Brequinar was acquired from MedChemExpress (MCE, Monmouth Junction, NJ, USA; Cat. No.: HY-108325).

### 2.2 Animals

The animal experiments were approved by the Animal Ethics Committee of Liuzhou Workers’ Hospital (Approval No.: KY202499). In the present study, the destabilization of the DMM technique was used to establish an OA model. To evaluate the therapeutic effects of Liubao tea, male C57BL/6 mice (6–8 weeks old) were randomly divided into four groups (n = 6 per group): Sham-operated control group, DMM group, DMM + low-dose Liubao Tea Extract (LPTE) group (100 mg/kg/day via intragastric gavage), and DMM + high-dose LPTE group (300 mg/kg/day via intragastric gavage). Samples were collected for analysis after 4 weeks of intervention.

For the DMM group: Mice were anesthetized, and hair over the surgical site was shaved off. The mice were placed in a supine position, and their left hind limbs were secured to maintain the knee joints flexed at 90°, followed by thorough disinfection of the surgical area. The skin was incised to expose the patellar ligament; a sharp scalpel was then used to dissect the joint capsule along the medial edge of the patellar ligament. The medial meniscus attaches to the tibial plateau via the medial meniscotibial collateral ligament (MMTL). The MMTL was transected, and the surgical wound was irrigated with sterile saline. The joint capsule was sutured with 7-0 surgical sutures, followed by skin closure. Topical amoxicillin ointment was applied to prevent wound infection.

For the Sham-operated control group, only the joint capsule was incised and then sutured in layers, with no additional interventions (i.e., no MMTL transection).

To investigate the role of pyrimidine synthesis inhibition in OA, a separate cohort of DMM-induced OA mice was treated with Brequinar (a specific *de novo* pyrimidine synthesis inhibitor). These mice were further subdivided into two groups: DMM + Vehicle group and DMM + Brequinar group (20 mg/kg Brequinar administered intragastrically [i.g.]).

### 2.3 Micro-CT analyses

Whole knee joints of mice were excised, and redundant tissues were carefully dissected away prior to fixation of the samples in paraformaldehyde. Subsequently, a NEMO-NMC200 micro-CT system was used to scan the samples at 50 kV and 800 μA. A 1.8 mm-thick region of the medullary cavity was selected, and 125 consecutive sections of the femoral epiphyseal plate were used for three-dimensional (3D) reconstruction. N-Recon software was employed for 3D image reconstruction, while Avatar software was used to quantify trabecular bone microstructural parameters, including trabecular thickness (Tb.Th), trabecular separation (Tb.Sp), trabecular number (Tb.N), and bone volume/tissue volume (BV/TV) ratio.

### 2.4 Hematoxylin-eosin and safranin O-fast green staining

Mouse knee joints were first fixed in 4% neutral buffered paraformaldehyde (PFA; Servicebio, Wuhan, China) at room temperature for 48 h, followed by decalcification in 12% ethylenediaminetetraacetic acid (EDTA, pH 7.4; Servicebio, Wuhan, China) at 4°C for 1 month (with the EDTA solution refreshed every 3 days to ensure consistent decalcification efficiency).

Following decalcification, samples were dehydrated through a graded ethanol series (70%, 80%, 90%, 95%, and 100% ethanol, 1 h each), cleared in xylene (twice, 30 min each), and then embedded in paraffin wax. A microtome (Leica, Wetzlar, Germany) was used to cut serial 4-μm-thick sagittal sections of the knee joint (centered on the medial femoral condyle and tibial plateau), which were then mounted on poly-L-lysine-coated glass slides. Hematoxylin and eosin (H&E; Servicebio, Wuhan, China) staining was used to measure cartilage surface thickness, while safranin O-fast green staining (Servicebio, Wuhan, China) was employed to evaluate cartilage matrix integrity (reflecting glycosaminoglycan content) following standard protocols. A digital slide scanning system (Pannoramic MIDI, 3DHISTECH, Budapest, Hungary) was used to acquire histological images at 20× magnification.

Modified Mankin Score: This score evaluates cartilage degeneration based on four parameters, with a total score ranging from 0 (normal cartilage) to 14 (severe degeneration): Cartilage structural integrity (0–4 points): 0 = intact cartilage surface and structure; 1 = superficial fibrillation without loss of cartilage thickness; 2 = fissures extending to the middle zone of cartilage; 3 = fissures reaching the deep zone of cartilage; 4 = complete loss of cartilage (exposing subchondral bone). Chondrocyte morphology (0–3 points): 0 = normal chondrocyte distribution (columnar arrangement in the deep zone); 1 = mild chondrocyte clustering (≤3 cells per cluster); 2 = moderate clustering (4–6 cells per cluster); 3 = severe clustering (>6 cells per cluster) or extensive chondrocyte loss. Safranin O staining intensity (0–4 points): 0 = intense, uniform staining (abundant glycosaminoglycans); 1 = mild reduction in staining intensity; 2 = moderate reduction (focal loss of staining); 3 = severe reduction (diffuse loss of staining); 4 = no detectable staining (complete glycosaminoglycan depletion). Tidemark integrity (0–3 points): 0 = intact, continuous tidemark; 1 = mild irregularity of the tidemark; 2 = partial disruption (focal breaks) of the tidemark; 3 = complete disruption (diffuse breaks) or disappearance of the tidemark. OARSI Score: This score quantifies the extent (Grade, G) and depth (Stage, S) of cartilage lesions: Grade (G, 0–4): Defined by the percentage of the cartilage surface involved: 0 = no visible damage; 1 = focal superficial lesions (<10% of the surface); 2 = multifocal superficial lesions (10%–50% of the surface); 3 = lesions involving ≥50% of the cartilage surface; 4 = lesions involving the entire cartilage surface. Stage (S, 0–3): Defined by the depth of cartilage damage: 0 = no damage; 1 = damage limited to the superficial zone (≤1/3 of cartilage thickness); 2 = damage extending to the middle zone (>1/3 to ≤2/3 of cartilage thickness); 3 = damage reaching the deep zone (>2/3 of cartilage thickness) or subchondral bone. The intraclass correlation coefficient (ICC) was used to calculate inter-observer agreement, with an ICC > 0.8 considered indicative of good consistency.

### 2.5 ELISA assay for cytokine detection

The serum levels of IL-6, IL-1β, and TNF-α were quantified using commercially available enzyme-linked immunosorbent assay (ELISA) kits, in accordance with the manufacturers’ instructions. The kits were purchased from Jianglai Biotechnology Co., Ltd. (China), with the following catalog numbers: IL-6 (Cat. No. JL20268), IL-1β (Cat. No. JL18442), and TNF-α (Cat. No. JL10484).

### 2.6 Gut microbiota analysis

PCR amplification and sequencing were performed according to the standard protocol provided by Shanghai OE Biotech Co., Ltd. Genomic DNA was used as the PCR template, with BARCODE-specific primers and Agencourt AMPure XP beads employed for the reaction. Primers targeting the V3-V4 region of the 16S rRNA gene were selected to ensure high amplification efficiency and accuracy, with the following sequences: forward primer (343F): TACGGRAGGCAGCAG; reverse primer (798R): AGGGTATCTAATCCT. PCR products of equal volume were pooled based on their concentrations and sequenced on the Illumina MiSeq PE300 platform. The resulting data were subsequently analyzed using the OE Biotech Cloud Platform (https://cloud.oebiotech.com/).

### 2.7 Fecal microbiota transplantation

Fresh fecal samples were collected from DMM-induced mice, either treated with Liubao tea or left untreated. Samples from each group were pooled to a total weight of 1 g and suspended in sterile PBS at a concentration of 0.125 g/mL. Recipient mice received daily intragastric administration of an antibiotic cocktail—consisting of vancomycin (50 mg/kg), neomycin sulfate (100 mg/kg), metronidazole (100 mg/kg), and ampicillin (100 mg/kg)—for 5 days prior to fecal microbiota transplantation (FMT). Following the antibiotic regimen, the mice were randomly assigned to two groups: the FMT-DMM group, which received fecal suspension from DMM-induced mice, and the FMT-LPTE group, which received fecal suspension from DMM-induced mice treated with Liubao tea. Each group was then administered 200 μL of the corresponding fecal suspension via intragastric gavage three times per week.

### 2.8 Non-targeted metabolomics analysis of mouse serum

Mouse serum samples (100 μL) were mixed with 400 μL of precooled methanol (-20°C) for protein precipitation. The mixture was vortexed vigorously for 3 min and then centrifuged at 14,000 × g for 15 min at 4°C. The resulting supernatant was transferred to a new 1.5 mL microcentrifuge tube, dried under a nitrogen gas stream at room temperature, and reconstituted in 100 μL of methanol. After vortexing and centrifugation (14,000 × g, 10 min, 4°C), 10 μL of the supernatant was injected into an Ultra Performance Liquid Chromatography-Quadrupole Time-of-Flight Mass Spectrometry (UPLC-Q-TOF MS) system for analysis. Chromatographic separation was achieved using a Waters ACQUITY UPLC BEH C18 column (2.1 × 50 mm, 1.7 μm) maintained at 40°C. The mobile phase consisted of 0.1% formic acid in water (solvent A) and acetonitrile (solvent B), with the following gradient program: 0–1.5 min: 95% A/5% B; 1.5–4.5 min: 75% A/25% B; 4.5–7 min: 70% A/30% B; 7–11 min: 45% A/55% B; 11–12 min: 15% A/85% B; 12–13.5 min: 5% A/95% B; 13.5–14 min: 95% A/5% B. The flow rate was 0.3 mL/min, and the injection volume was 1 μL. Mass spectrometry was performed with an electrospray ionization (ESI) source operating in both positive and negative ion modes. Key MS parameters were configured as follows: mass range, m/z 100–1000; spray voltage, 3.5 kV (positive) and 3.2 kV (negative); sheath gas flow rate, 40 arbitrary units (arb); auxiliary gas flow rate, 5 arbitrary units (arb); ion transfer tube temperature, 320°C; auxiliary gas heater temperature, 350°C; collision energy, 20–40 eV (for MS/MS). Raw data were processed using Progenesis QI software (Waters Corporation) for peak alignment, denoising, and normalization. Metabolites were identified by matching accurate mass, retention time, and MS/MS fragmentation patterns with the Human Metabolome Database (HMDB), MetLin, and an in-house standard library. Multivariate statistical analyses, including principal component analysis (PCA) and orthogonal partial least squares-discriminant analysis (OPLS-DA), were performed using SIMCA-P 14.1 software to screen for differential metabolites (variable importance in projection (VIP) > 1 and p < 0.05 by Student’s t-test).

### 2.9 UPLC-MS/MS analysis of metabolites in Liubao tea

Liubao tea samples were freeze-dried using a Scientz-100F lyophilizer (Scientz Biotechnology Co., Ltd., Ningbo, China) and ground into a fine powder with an MM 400 grinder (Retsch GmbH, Haan, Germany) at 30 Hz for 1.5 min. Approximately 50 mg of sample powder was accurately weighed and mixed with 1200 μL of pre-cooled 70% (v/v) methanolic aqueous solution (pre-cooled to -20°C) containing internal standards (with the volume scaled proportionally for samples < 50 mg). The mixture was vortexed for 30 s every 30 min (total of 6 times), centrifuged at 12,000 rpm (13,800 × g) at 4°C for 3 min, and the supernatant was filtered through a 0.22 μm hydrophilic polyvinylidene fluoride (PVDF) microporous membrane before being stored in amber injection vials for UPLC-MS/MS analysis. UPLC analysis was performed on an ExionLC™ AD system (Sciex LLC, Framingham, MA, USA) equipped with an Agilent ZORBAX SB-C18 column (1.8 µm, 2.1 mm × 100 mm; Agilent Technologies, Santa Clara, CA, USA). The mobile phase consisted of solvent A (0.1% (v/v) formic acid in ultrapure water) and solvent B (0.1% (v/v) formic acid in acetonitrile), with a gradient program as follows: 95% A/5% B (0 min) → 5% A/95% B (9 min, held for 1 min) → 95% A/5% B (10.1 min, held for 2.9 min).

The flow rate was 0.35 mL/min, the column temperature was 40°C, and the injection volume was 2 μL (with the needle washed using 50% methanol between injections). MS/MS detection was conducted on an ESI-QTRAP mass spectrometer (Sciex LLC, Framingham, MA, USA) with the following parameters: source temperature, 500°C; ion spray voltage, 5500 V (positive mode)/-4500 V (negative mode); gas curtain (CUR), gas 1 (GSI), and gas 2 (GSII) set to 25, 50, and 60 psi, respectively; collision-activated dissociation (CAD) at high level. Targeted multiple reaction monitoring (MRM) scans were performed with medium-pressure collision gas (nitrogen), and the declustering potential (DP) and collision energy (CE) were optimized individually for each target metabolite.

### 2.10 Network pharmacology

To explore the active components, potential targets, and key pathways of Liubao tea in the treatment of OA, a network pharmacology analysis was performed as follows. Briefly, one Liubao tea sample was subjected to metabolomic analysis using a UPLC-MS/MS platform. The Simplified Molecular-Input Line-Entry System (SMILES) strings of the identified metabolites were retrieved from the PubChem database (https://pubchem.ncbi.nlm.nih.gov/). The chemical constituents of Liubao tea were screened in silico for drug-likeness and pharmacokinetic properties using the ADMETlab 2.0 platform, consistent with previous reports (20, 21). Briefly, potential bioactive compounds were identified by applying a stringent multi-criteria filter. They were required to comply with Lipinski’s Rule of Five: molecular weight ≤ 500, hydrogen-bond donors ≤ 5, hydrogen-bond acceptors ≤ 10, logP ≤ 5, and rotatable bonds ≤ 10. In addition, they had to meet key ADMET thresholds, including predicted human oral bioavailability (F20%) ≥ 0.7, low blood-brain barrier permeability (≤ 0.7), low hERG blockade risk (≤ 0.7), low hepatotoxicity potential (≤ 0.7), and a topological polar surface area (TPSA) ≤ 140 Å². OA-pathogenic genes were retrieved from the DisGeNet database (https://www.disgenet.org/). We searched for all OA-related diseases and their corresponding genes, and genes supported by literature reports were defined as OA-pathogenic genes. The overlapping targets between the active components of Liubao tea and OA were identified using Venn diagram analysis. A “Liubao tea-OA” network was constructed with these intersection targets using Cytoscape 3.9.1.

### 2.11 GO and KEGG analysis

Gene Ontology (GO) and Kyoto Encyclopedia of Genes and Genomes (KEGG) enrichment analyses were performed to annotate the functional roles and potential signaling pathways of the overlapping genes. For GO enrichment analysis, the DAVID database (https://davidbioinformatics.nih.gov/) was utilized with the following parameters: species was restricted to Homo sapiens, gene identifier was set as official gene symbol, and enrichment categories included Biological Process (BP), Cellular Component (CC), and Molecular Function (MF) (GOTERM_BP_DIRECT, GOTERM_CC_DIRECT, GOTERM_MF_DIRECT). Terms with a false discovery rate (FDR) < 0.01 were considered significantly enriched, and the top enriched terms in each category were selected for visualization. KEGG pathway enrichment analysis was conducted using the KEGG database (https://www.genome.jp/kegg/) or DAVID database. The same gene list and species setting as GO analysis were applied. Significantly enriched pathways were filtered with FDR < 0.01, and key pathways were summarized for subsequent analysis. All visualization plots (e.g., bar charts for GO terms, bubble charts for KEGG pathways) were generated using R software (version 4.0.2).

### 2.12 Construction of the protein-protein interaction network for OA targets

The predicted targets obtained from the intersection were imported into the STRING online database (https://string-db.org), with the species set to Homo sapiens. A protein-protein interaction (PPI) network of OA-related targets was constructed with a confidence score cutoff of > 0.7. Subsequently, the network data were imported into Cytoscape 3.7.1, and these intersecting drug and disease targets were analyzed using the BisoGenet and CytoNCA plugins. Hub targets of the network were identified through topological analysis based on three key topological parameters: Degree, Betweenness Centrality, and Closeness Centrality. The size of the network nodes was set based on the Degree value.

### 2.13 Compound–target–pathological gene network construction

The construction of the C-T-P network was performed as described in previous studies (22, 23). The potential targets of the active compounds of Liubao tea were predicted by using the following web servers: Similarity Ensemble Approach (SEA) Search Server (https://sea.bkslab.org/), CDD Vault HitFinder (https://www.collaborativedrug.com/), and SwissTargetPrediction (http://www.swisstargetprediction.ch/index.php). The union set of the prediction results is defined as the potential targets. Pathological genes for osteoarthritis (OA) were searched and downloaded from the DisGeNet database (https://disgenet.com/). We searched all OA-related diseases and their corresponding genes. Genes with literature reports were defined as pathological genes for OA. The intersection of target genes and OA pathological genes is defined as essential common genes. We retrieved the interactions between target genes and OA pathological genes and extracted protein-protein interactions that were reported in the literature. Combining the previous Liubao tea compound–target prediction results, the compound–target–pathological gene (C-T-P) network was constructed and visualized using Gephi software (https://gephi.org/).

### 2.14 Molecular docking

High-resolution crystal structures of the hub protein targets were retrieved from the RCSB Protein Data Bank (RCSB PDB; https://www.rcsb.org/) and selected as molecular docking receptors. PyMOL software was used to process the proteins, including the removal of water molecules and phosphate groups, and the processed structures were saved in PDB format. AutoDock Vina 1.5.6 was employed for molecular docking to investigate protein-ligand interactions. Prior to docking, structural preprocessing of the proteins and small-molecule ligands was performed using AutoDock Tools: hydrogen atoms were added to the proteins and water molecules were removed; for the small-molecule ligands, hydrogenation was conducted and torsion angles were defined. Subsequently, the coordinates of the docking box were determined. The optimal conformations of the molecular simulations were ultimately obtained by comparing the docking scores. Discovery Studio 2019 and PyMOL software were utilized to visualize the 2D interaction diagrams between the test compounds and the key amino acid residues of the target proteins.

### 2.15 Statistical analysis

Data are presented as the mean ± standard error of the mean (SEM). Differences between two groups were assessed using Student’s t-test, while those among multiple groups were analyzed using one-way analysis of variance (ANOVA). A p-value < 0.05 was considered statistically significant. All statistical analyses were performed using GraphPad Prism 8.0 and SPSS 29.0.

## 3 Results

### 3.1 Liubao tea ameliorates DMM-induced OA in a mouse model

**Figure 1:**
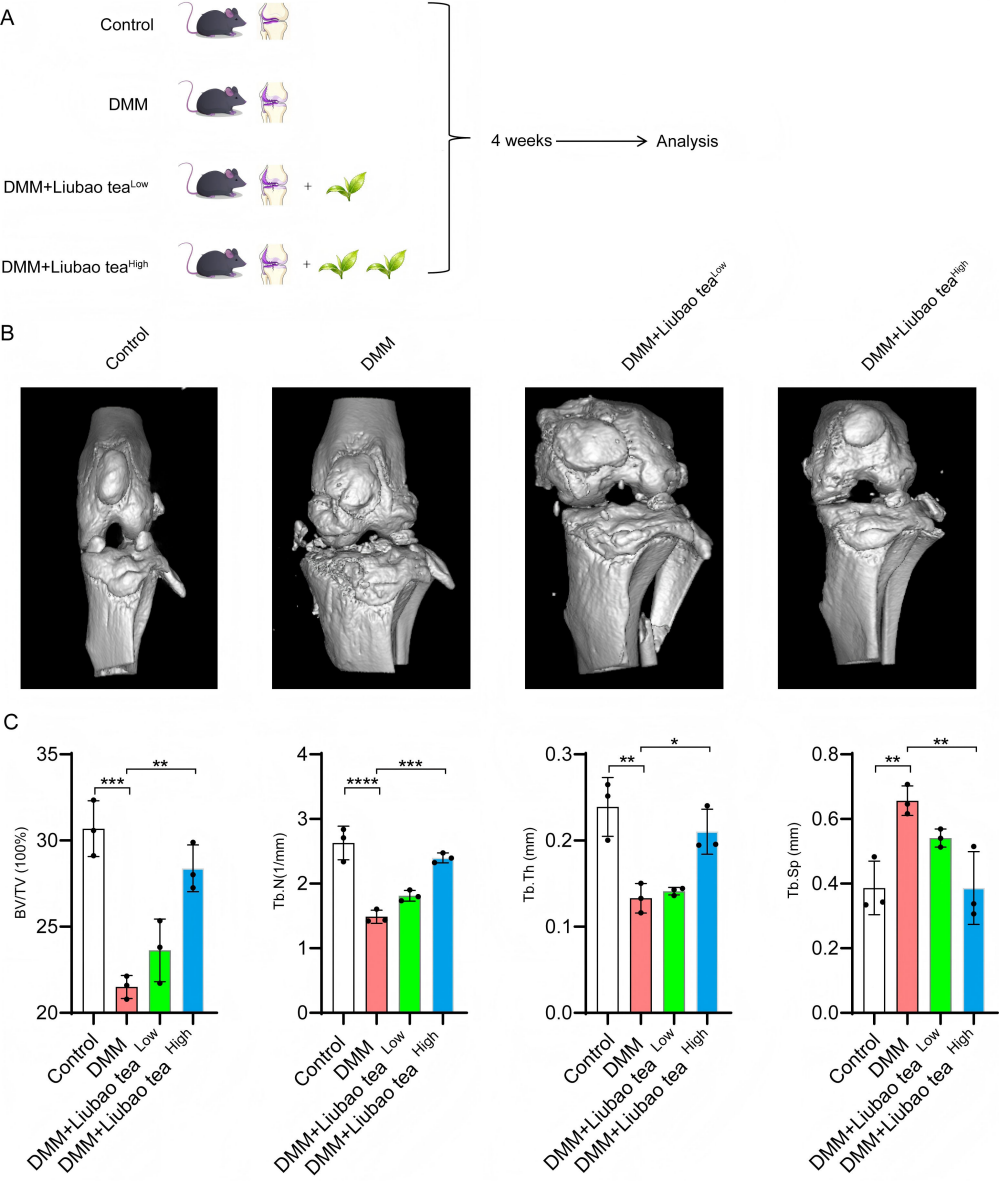
Micro-CT analysis of mouse knee joints. **(A)** Schematic diagram of the animal experimental design. **(B)** Micro-CT images of knee joints from the control, DMM, DMM + low-dose Liubao tea, and DMM + high-dose Liubao tea groups. **(C)** Quantitative analysis of subchondral bone microstructural parameters, including BV/TV, Tb.N, Tb.Th, and Tb.SP. One-way analysis of variance (ANOVA) followed by Sidak’s correction for multiple comparisons was used for **(C)**. All data are presented as mean ± standard error of the mean (SEM). ****p < 0.0001, ***p < 0.001, **p < 0.01, *p < 0.05.

**Figure 2:**
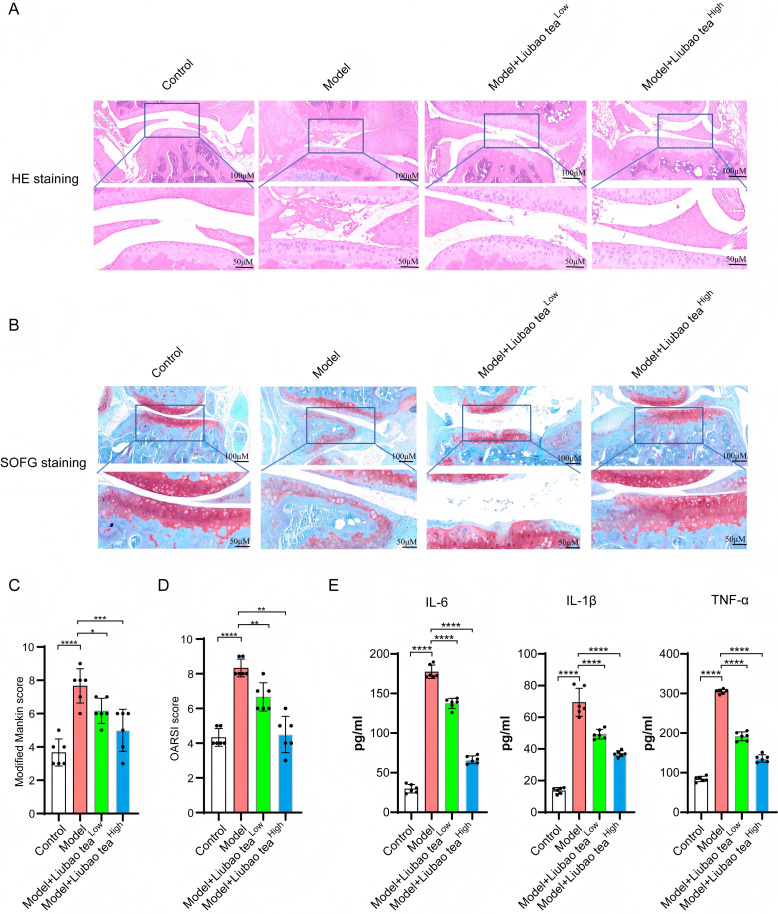
Liubao tea treatment alleviates osteoarthritis pathological progression and reduces inflammatory levels. **(A, B)** Representative histological images of knee joint cartilage stained with H&E) **(A)** and SOFG **(B)**. **(C)** Modified Mankin scores for each group. **(D)** OARSI scores for the four groups of mice. **(E)** Levels of IL-6, IL-1β, and TNF-α in serum from each group of mice measured by enzyme-linked immunosorbent assay (ELISA). One-way ANOVA followed by Sidak’s correction for multiple comparisons was used for **(C–E)**. All data are presented as mean ± SEM. ****p < 0.0001, ***p < 0.001, **p < 0.01, *p < 0.05.

To evaluate the therapeutic potential of Liubao tea against OA, a mouse model was established via destabilization of the DMM surgery (**Figure 1A**). Following Liubao tea intervention, micro-CT imaging revealed notable improvements in the knee joints of OA mice, including reduced joint space narrowing, smoother articular surfaces, and diminished osteophyte formation (**Figure 1B**). Quantitative assessment of subchondral bone microstructure indicated that Liubao tea significantly increased BV/TV, Tb.N, and Tb.Th, while decreasing Tb.Sp (**Figure 1C**). Histopathological analysis of knee joint sections stained with H&E and SOFG was performed to examine cartilage morphology across groups (**Figures 2A, B**). In sham-operated control mice, the cartilage structure and tidemark remained intact, with chondrocytes arranged uniformly. By contrast, the DMM group exhibited uneven and severely eroded cartilage margins, chondrocyte loss, cartilage layer thinning, and reduced or absent Safranin O staining. Liubao tea treatment mitigated these pathological changes, alleviating cartilage damage, restoring chondrocyte numbers, and improving cartilage architecture. Consistently, both the Modified Mankin scores and the OARSI scores were significantly reduced following Liubao tea administration (**Figures 2C, D**). Furthermore, serum analysis demonstrated markedly elevated levels of the pro-inflammatory cytokines IL-6, IL-1β, and TNF-α in DMM-induced mice, all of which were significantly suppressed after Liubao tea treatment (**Figure 2E**). Taken together, these findings indicate that Liubao tea alleviates joint structural deterioration and attenuates inflammatory responses in a mouse model of DMM-induced OA.

### 3.2 Effects of Liubao tea on gut microbiota in DMM-induced OA

**Figure 3:**
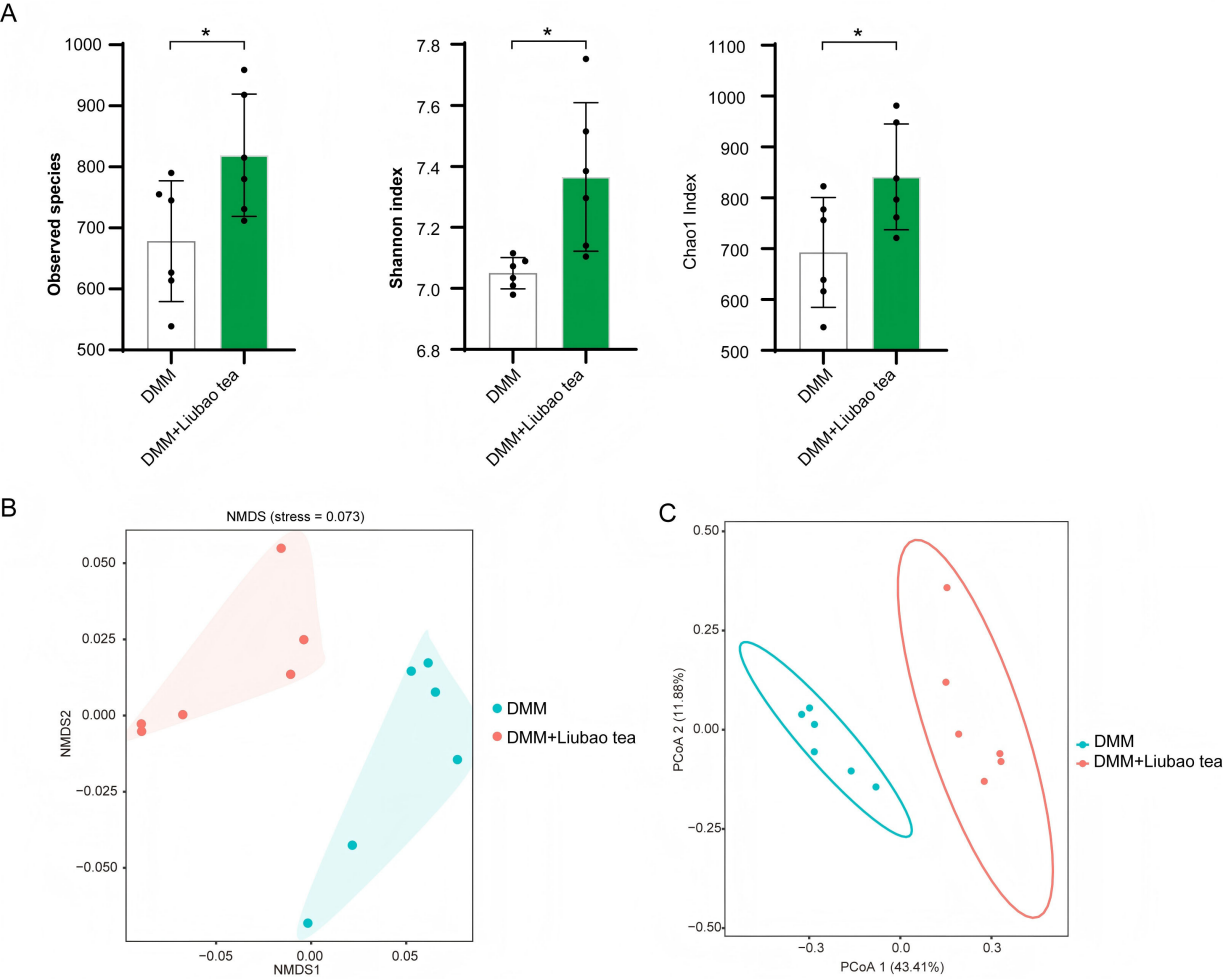
Effects of Liubao tea on gut microbiota in osteoarthritis mice. **(A)** α-diversity analyzed by observed species, Shannon, and Chao1 indices. **(B)** NMDS analysis based on Bray-Curtis dissimilarity to visualize differences in gut microbiota community structure between the DMM and DMM + Liubao tea groups. **(C)** PCoA between the DMM and DMM + Liubao tea groups. Student’s t-test was used for **(A)**. All data are presented as mean ± SEM. *p < 0.05.

**Figure 4:**
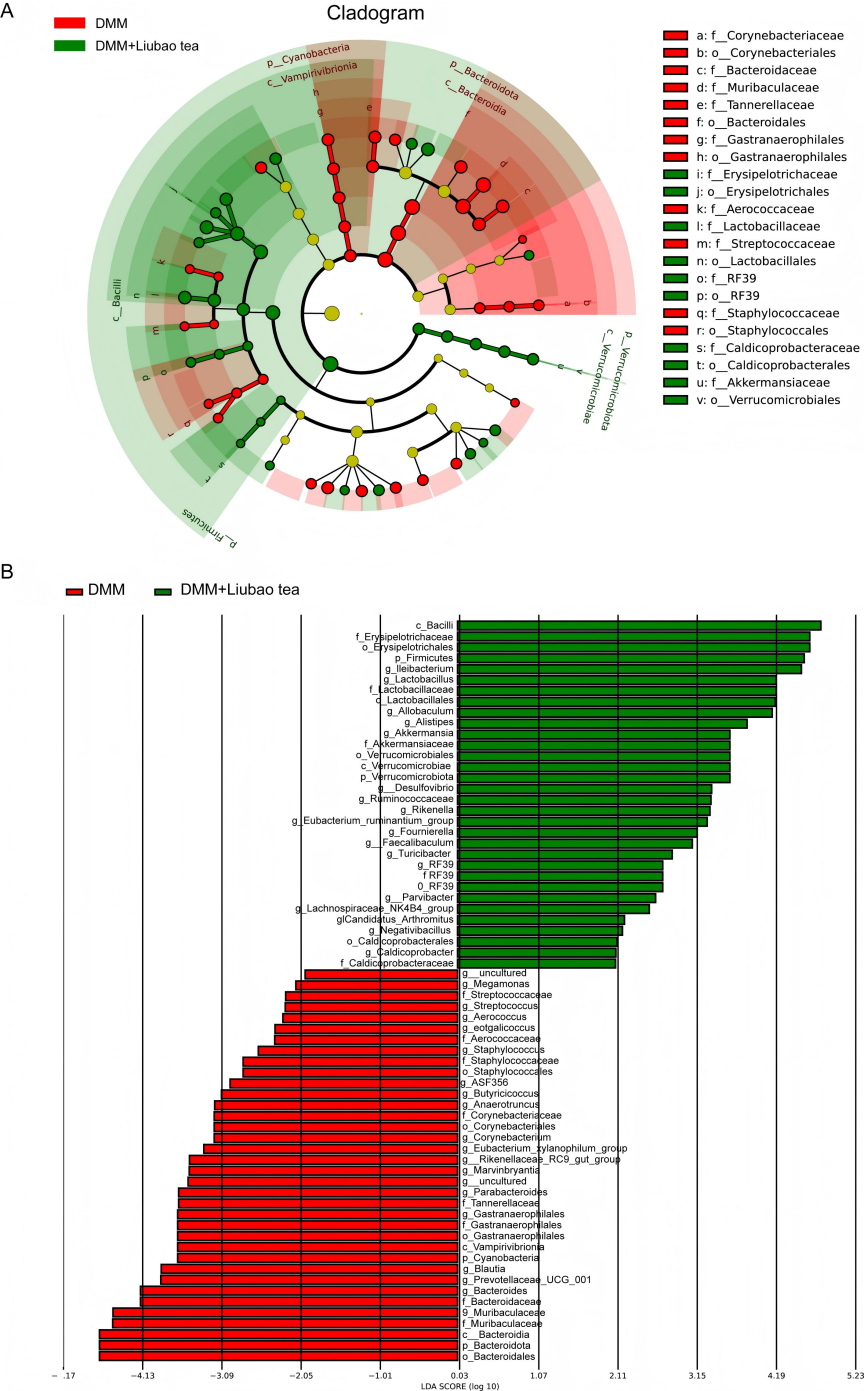
LEfSe **(A)** and phylogenetic tree-based **(B)** analysis were conducted to identify differentially abundant bacterial taxa among the groups.

Previous studies have indicated that Liubao tea can modulate gut microbiota and ameliorate multiple diseases (15–17). Therefore, this study further investigated whether Liubao tea regulates the gut microbiota in a mouse model of DMM-induced osteoarthritis. The results demonstrated that Liubao tea treatment increased the α-diversity of the gut microbiota in OA mice, as reflected by higher values of the Observed Species, Shannon, and Chao1 indices (**Figure 3A**). β-diversity analysis using non-metric multidimensional scaling (NMDS) and principal coordinate analysis (PCoA) revealed a distinct separation in microbial community structure between the DMM-induced OA group and the Liubao tea-treated group, indicating substantial shifts in gut microbiota composition (**Figures 3B, C**). In addition, Linear Discriminant Analysis Effect Size (LEfSe) and phylogenetic tree-based analysis were conducted to identify differentially abundant bacterial taxa among the groups (**Figures 4A, B**). Collectively, these findings indicate that Liubao tea treatment induces substantial alterations in the gut microbiota of mice with DMM-induced OA.

### 3.3 FMT from Liubao tea-treated mice ameliorates DMM-induced OA in mice

**Figure 5:**
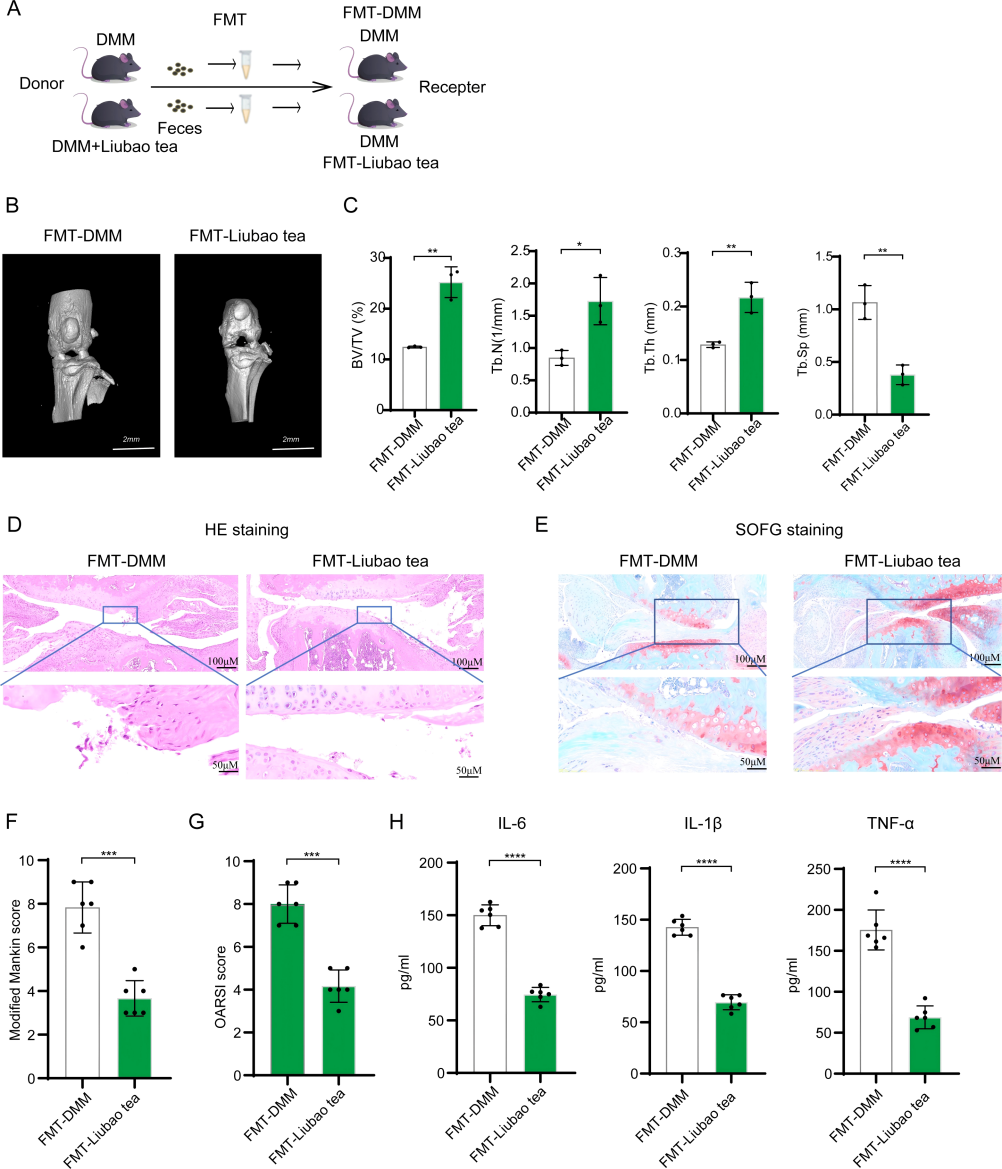
Effect of fecal microbiota transplantation (FMT) from Liubao tea-treated mice on osteoarthritis. **(A)** Graphical abstract of the FMT study. **(B)** Micro-CT images of knee joints from the FMT-DMM and FMT-Liubao tea groups. **(C)** Quantitative analysis of subchondral bone microstructural parameters, including BV/TV, Tb.N, Tb.Th, and Tb.SP. **(D)** Representative histological images of knee joint cartilage stained with H&E and SOFG. **(E)** Modified Mankin scores for each group. **(F)** OARSI scores for the two groups of mice. **(G)** Levels of IL-6, IL-1β, and TNF-α in serum from each group of mice measured by ELISA. **(H)** The serum levels of pro-inflammatory cytokines (IL-6, IL-1β, and TNF-α) in mice from each group were determined by ELISA. Student’s t-test was used for **(C, E, F, G)**. All data are presented as mean ± SEM. ****p < 0.0001, ***p < 0.001, **p < 0.01, *p < 0.05.

Previous studies have established that FMT can ameliorate osteoarthritis in mice (24). Building on the observed modulatory effect of Liubao tea on the gut microbiota of DMM-induced OA mice, we further examined whether FMT from Liubao tea-treated donors confers therapeutic benefits in DMM-induced OA mice. Recipient DMM-induced OA mice received fecal microbiota derived from Liubao tea-treated donors (**Figure 5A**). Micro-CT analysis of knee joints showed that FMT resulted in a wider joint space, a smoother articular surface, and reduced osteophyte formation (**Figure 5B**). Consistent with these observations, FMT significantly increased BV/TV, Tb.N, and Tb.Th, while significantly reducing Tb.Sp (**Figure 5C**). Histological evaluation using H&E and SOFG staining revealed that FMT alleviated cartilage damage and improved cartilage architecture (**Figures 5D, E**). Accordingly, both the Modified Mankin scores and OARSI scores were significantly reduced in the FMT group (**Figures 5F, G**). Moreover, serum levels of the pro-inflammatory cytokines IL-6, IL-1β, and TNF-α were markedly reduced following FMT (**Figure 5H**). Collectively, these findings demonstrate that transplantation of fecal microbiota from Liubao tea-treated mice alleviates DMM-induced OA in recipient mice.

### 3.4 Liubao tea modulates serum metabolites in DMM-induced OA mice

**Figure 6:**
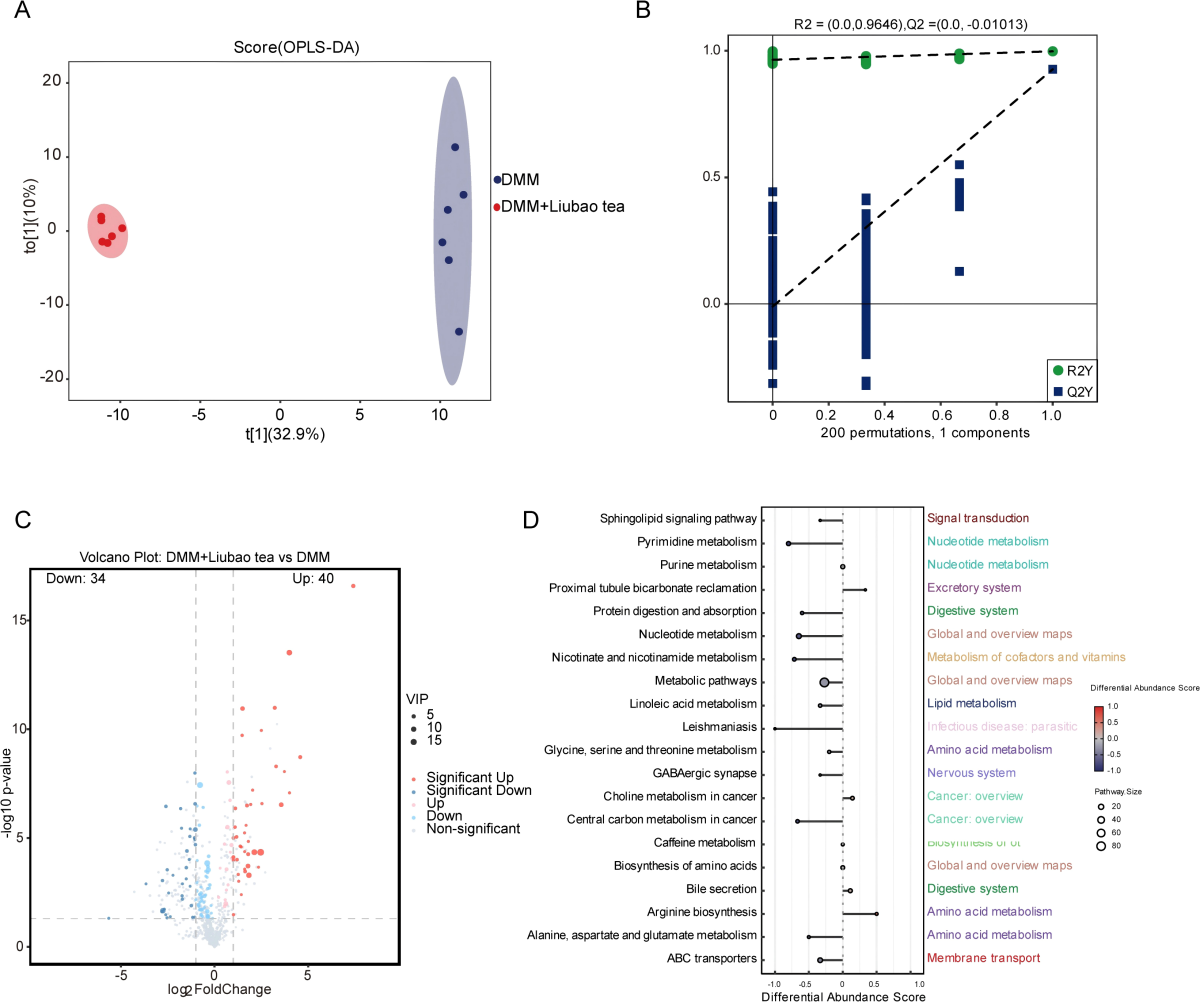
Serum metabolomics analysis of the DMM and DMM + Liubao tea groups. **(A, B)** OPLS-DA score plot **(A)** and permutation test **(B)** for the comparison between the DMM and DMM + Liubao tea groups. **(C)** Volcano plot comparing the DMM group and the DMM + Liubao tea group; criteria for identifying significant differences were defined as VIP > 1.0, FC > 2 or < 0.5, and p < 0.05. **(D)** Kyoto Encyclopedia of Genes and Genomes (KEGG) pathway enrichment differential abundance score plot.

To further investigate the effects of Liubao tea on serum metabolites in DMM-induced OA mice, we performed untargeted metabolomic analysis on serum samples. The metabolomic data were subjected to OPLS-DA. Score plots revealed clear separation among the groups, confirming distinct metabolic profiles (**Figure 6A**). Permutation tests confirmed the validity of the OPLS-DA models, with R² and Q² values showing the expected trends, indicating robust stability and reliable predictability of the models (**Figure 6B**). Volcano plots were used to identify differential metabolites based on the following criteria: VIP > 1.0, FC > 2 or < 0.5, and p < 0.05. Compared with the DMM-induced OA group, 40 metabolites were upregulated and 34 were downregulated in the Liubao tea-treated DMM-induced OA mice (**Figure 6C**). KEGG pathway enrichment analysis was visualized using a differential abundance (DA) score plot, which highlighted pyrimidine metabolism as the most significantly downregulated pathway (**Figure 6D**). Collectively, these results indicate that Liubao tea intervention markedly alters the serum metabolome in DMM-induced OA mice.

### 3.5 Inhibition of endogenous pyrimidine synthesis ameliorates DMM-induced OA

**Figure 7:**
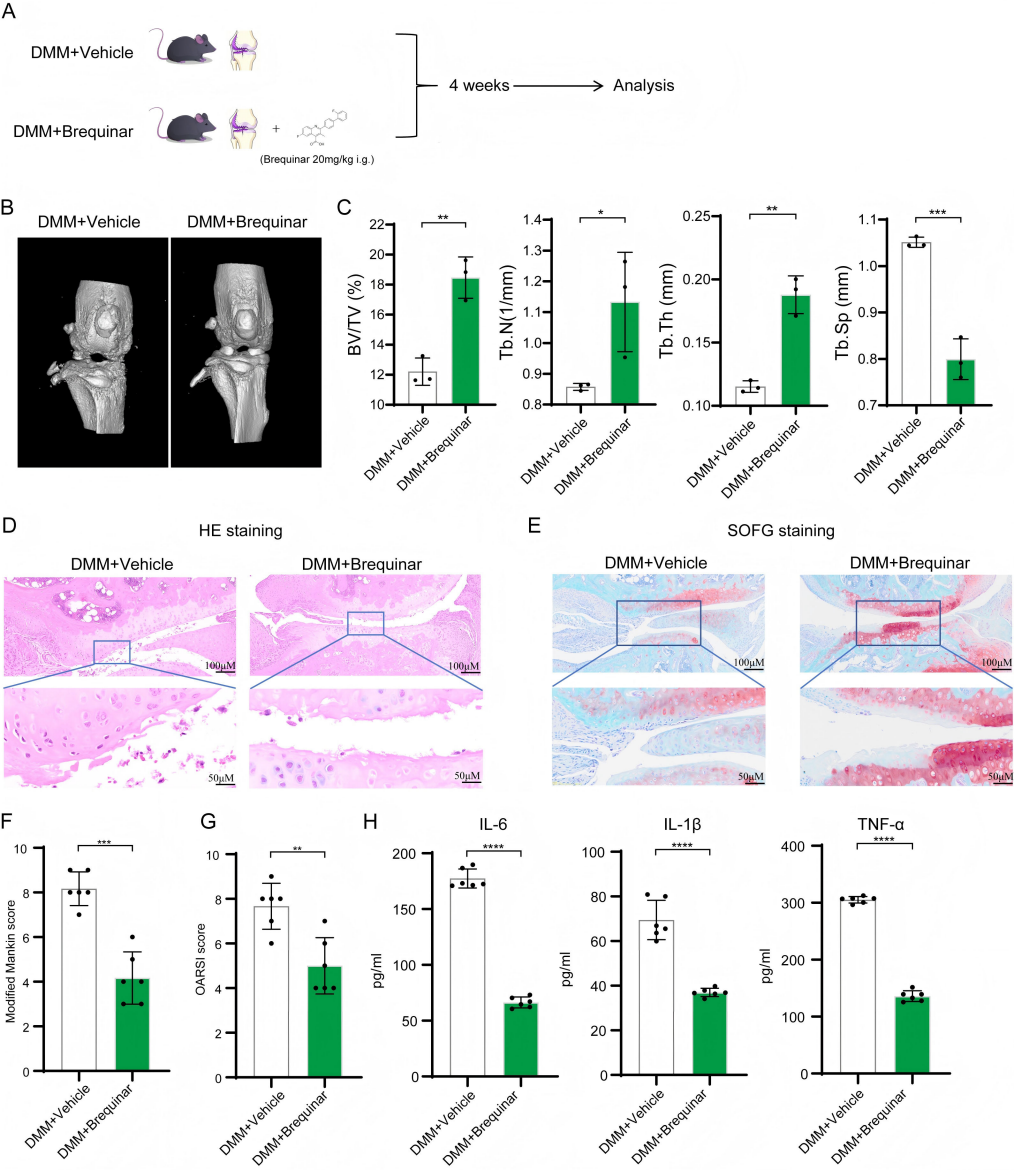
Effects of Brequinar on osteoarthritis. **(A)** Graphical abstract of Brequinar treatment. **(B)** Micro-CT images of knee joints from the DMM + Vehicle and DMM + Brequinar groups. **(C)** Quantitative analysis of subchondral bone microstructural parameters, including BV/TV, Tb.N, Tb.Th, and Tb.SP. **(D)** Representative histological images of knee joint cartilage stained with H&E and SOFG. **(E)** Modified Mankin scores for each group. **(F)** OARSI scores for the two groups of mice. **(G)** Levels of IL-6, IL-1β, and TNF-α in serum from each group of mice measured by ELISA. **(H)** The serum levels of pro-inflammatory cytokines (IL-6, IL-1β, and TNF-α) in mice from each group were determined by ELISA. Student’s t-test was used for **(C, E–G)**. All data are presented as mean ± SEM. ****p < 0.0001, ***p < 0.001, **p < 0.01, *p < 0.05.

Metabolomic findings revealed that Liubao tea suppressed the pyrimidine metabolism pathway. To determine whether Liubao tea alleviates DMM-induced OA by regulating pyrimidine metabolism, we treated DMM-induced OA mice with Brequinar—a specific inhibitor of *de novo* pyrimidine synthesis (**Figure 7A**). Micro-CT analysis indicated that Brequinar administration markedly improved knee joint pathology in DMM-induced OA mice (**Figure 7B**). Quantitative evaluation showed that Brequinar significantly increased BV/TV, Tb.N, and Tb.Th, while significantly reducing Tb.Sp (**Figure 7C**). Correspondingly, H&E and SOFG staining revealed that Brequinar mitigated inflammatory infiltration and articular cartilage degradation (**Figures 7D, E**). Both the Modified Mankin scores and OARSI scores were significantly lower in the Brequinar-treated group compared with the DMM-induced OA group (**Figures 7F, G**). Additionally, Brequinar treatment significantly decreased serum levels of the pro-inflammatory cytokines IL-6, IL-1β, and TNF-α (**Figure 7H**). Collectively, these data demonstrate that inhibition of pyrimidine synthesis ameliorates DMM-induced OA, implying that the therapeutic effect of Liubao tea on DMM-induced OA may be mediated, at least in part, through suppression of pyrimidine metabolism.

### 3.6 Composition analysis of Liubao tea and identification of its potential therapeutic targets for OA via network pharmacology

**Figure 8:**
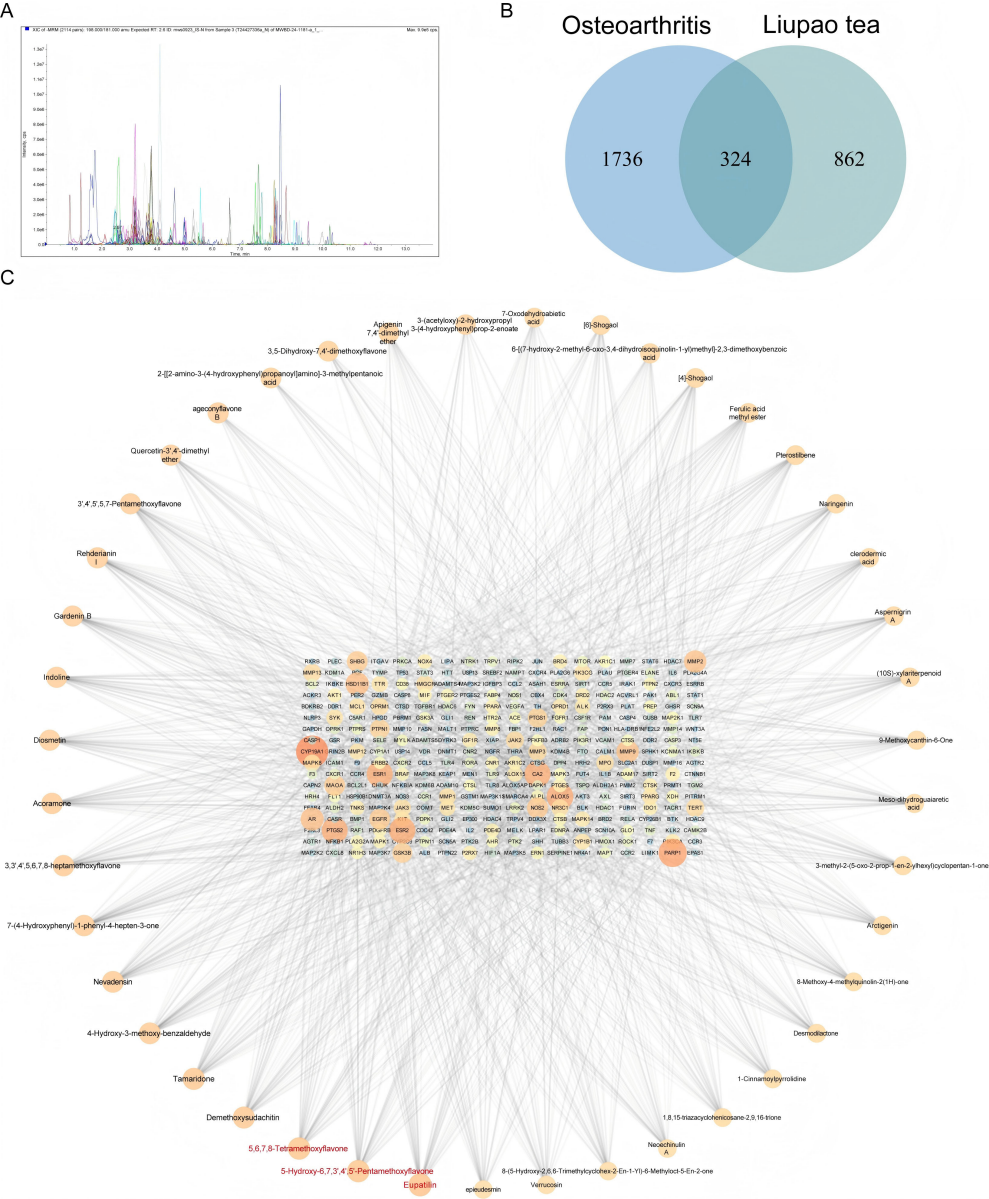
Composition analysis of Liubao tea and network pharmacology analysis. **(A)** Mass spectrogram of Liubao tea in positive ion mode. **(B)** Venn diagram illustrating the overlap between Liubao tea-related targets and osteoarthritis-related targets. **(C)** Component-Target Network constructed using Cytoscape software.

The chemical components of Liubao tea were initially characterized using ultra-performance liquid chromatography-tandem mass spectrometry (UPLC-MS/MS), with the resulting positive ion flow chromatograms shown in **Figure 8A**. Using the UPLC-MS/MS platform and an in-house database, a total of 1989 metabolites were identified. These metabolites were primarily categorized into flavonoids (571 species), phenolic acids (290 species), quinones (30 species), and lignans and coumarins (123 species), among other classes (see **Supplementary Table 1**). In silico screening of the chemical constituents of Liubao tea for drug-likeness and pharmacokinetic properties was conducted using the ADMETlab 2.0 platform. Potential bioactive compounds were identified through a stringent multi-criteria filter that required compliance with Lipinski’s Rule of Five, yielding a total of 1186 targets corresponding to these bioactive components.

**Figure 9:**
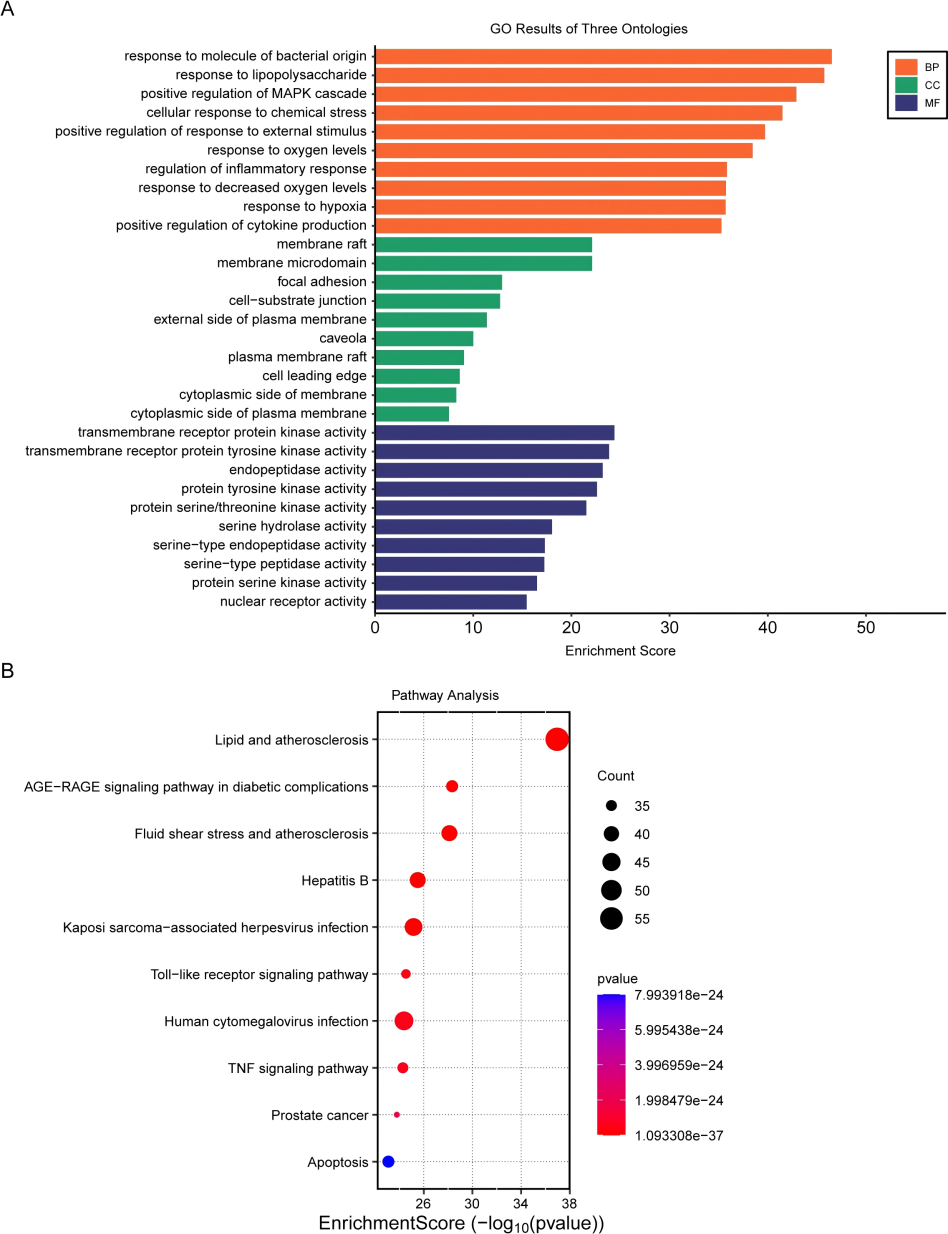
GO and KEGG analysis. **(A)** Top enriched GO terms in Biological Process, Cellular Component, and Molecular Function categories for overlapping genes. **(B)** KEGG pathway enrichment analysis of the overlapping targets using the Metascape database, with the top 10 most significantly enriched pathways selected for visualization.

**Figure 10:**
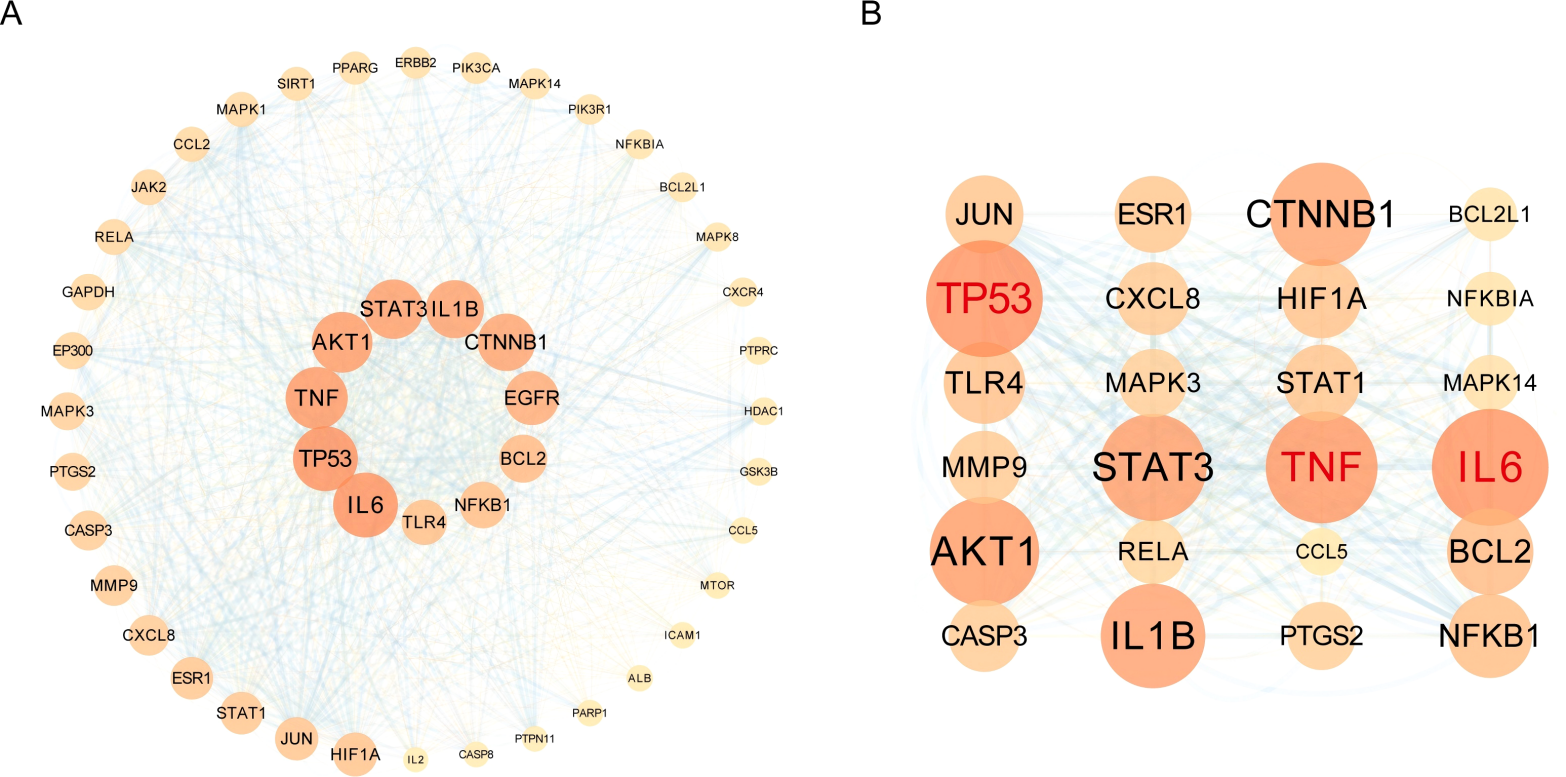
Liubao tea‑associated PPI network of OA targets. **(A)** Liubao tea‑related osteoarthritis targets were imported into Cytoscape software. Hub targets of the network were identified via topological analysis based on three key parameters: Degree, Betweenness Centrality, and Closeness Centrality, with node sizes scaled according to Degree values. **(B)** Three rounds of topological analyses using the same three parameters were further performed to construct the hub target protein network.

Subsequently, Venn diagram analysis was performed to identify overlapping targets between the bioactive component targets of Liubao tea and OA-related genes, resulting in 324 intersection genes (**Figure 8B**). Based on the screened bioactive components and their corresponding targets, a component-target network was constructed; compounds with a Degree value > 30 and their matched targets were visualized (**Figure 8C**). The GO and KEGG analyses of overlapping genes are presented in **Figure 9**. Furthermore, a PPI network of the 324 intersection targets was constructed using the STRING online database, containing 324 nodes and 810 interaction edges. Thereafter, the drug targets and network targets were imported into Cytoscape 3.7.1 for mapping and screening; three rounds of topological analyses based on Degree, Betweenness Centrality, and Closeness Centrality were performed to construct the hub target protein network (**Figure 10A**). This analysis ultimately identified TP53, IL6, and TNF as the top 3 core hub targets of Liubao tea for OA treatment (**Figure 10B**).

**Figure 11:**
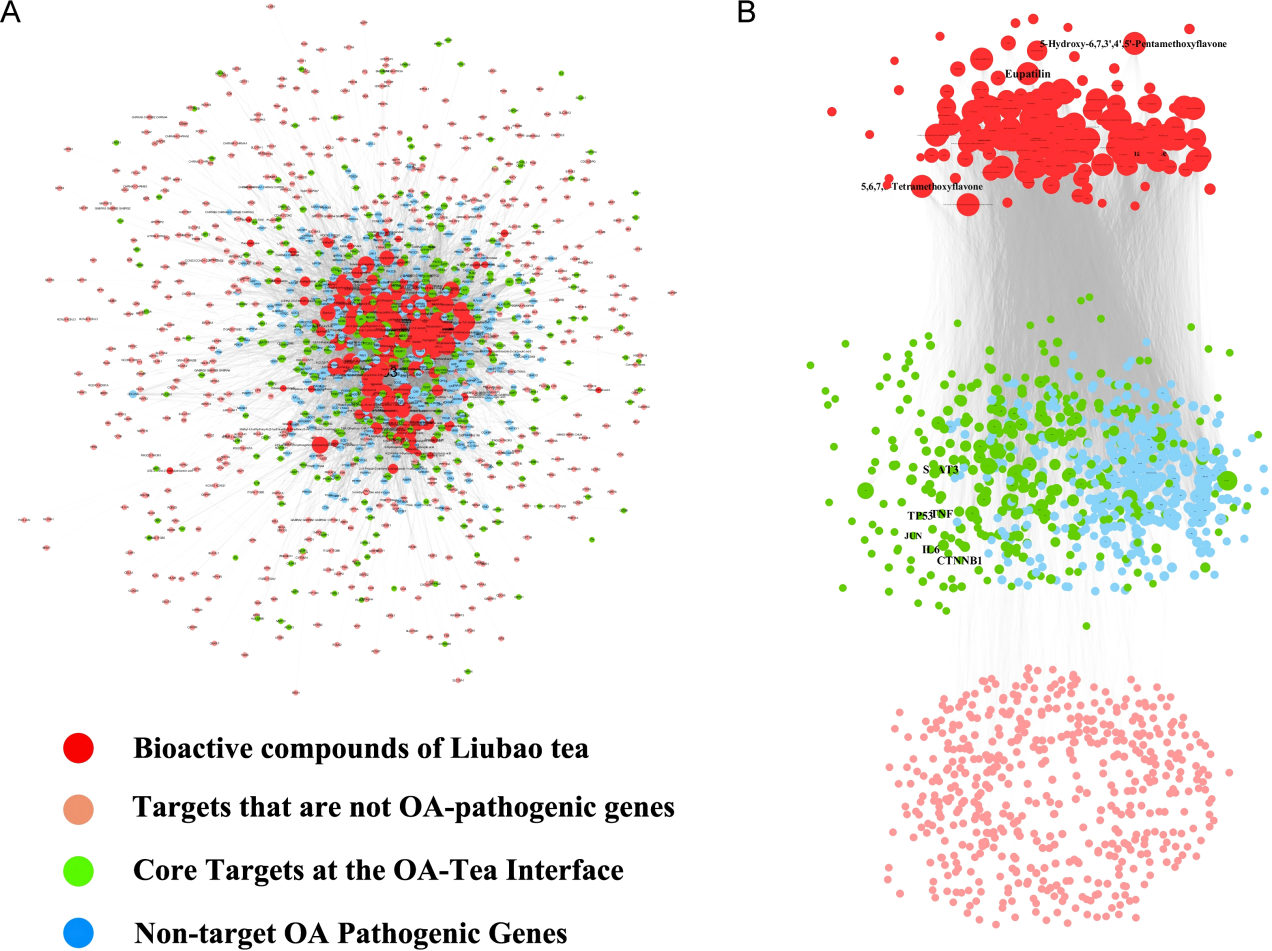
The original **(A)** and optimized **(B)** C-T-P network. The red, pink, blue, and green points indicate bioactive compounds of Liubao tea, targets that are not OA-pathogenic genes, non-target OA pathogenic genes, and core targets at the OA-tea interface, respectively. Compounds and genes with high degrees were highlighted and labeled.

Subsequently, we integrated the compound-target network with the PPI network to construct a compound-target-protein (C-T-P) network. The original C-T-P network contained 10,146 compound-target pairs (**Figure 11A**). Within this network, 324 proteins functioned as both target proteins and pathogenic genes, which were defined as core shared proteins. Subsequently, Cytoscape software was employed for network optimization and screening of bioactive compounds, generating an optimized C-T-P network (**Figure 11B**). This optimized network comprised 7,823 compound-target pairs, involving 169 compounds, 1,186 predicted targets and 2,060 disease-related genes. All core shared proteins were retained in the optimized network. In the interaction network, compounds including eupatilin, 5,6,7,8-tetramethoxyflavone, 5-hydroxy-6,7,3’,4’,5’-pentamethoxyflavone, demethoxysudachitin and tamaridone exhibited relatively high connectivity degrees.

### 3.7 Molecular docking of candidate compounds and target proteins

**Figure 12:**
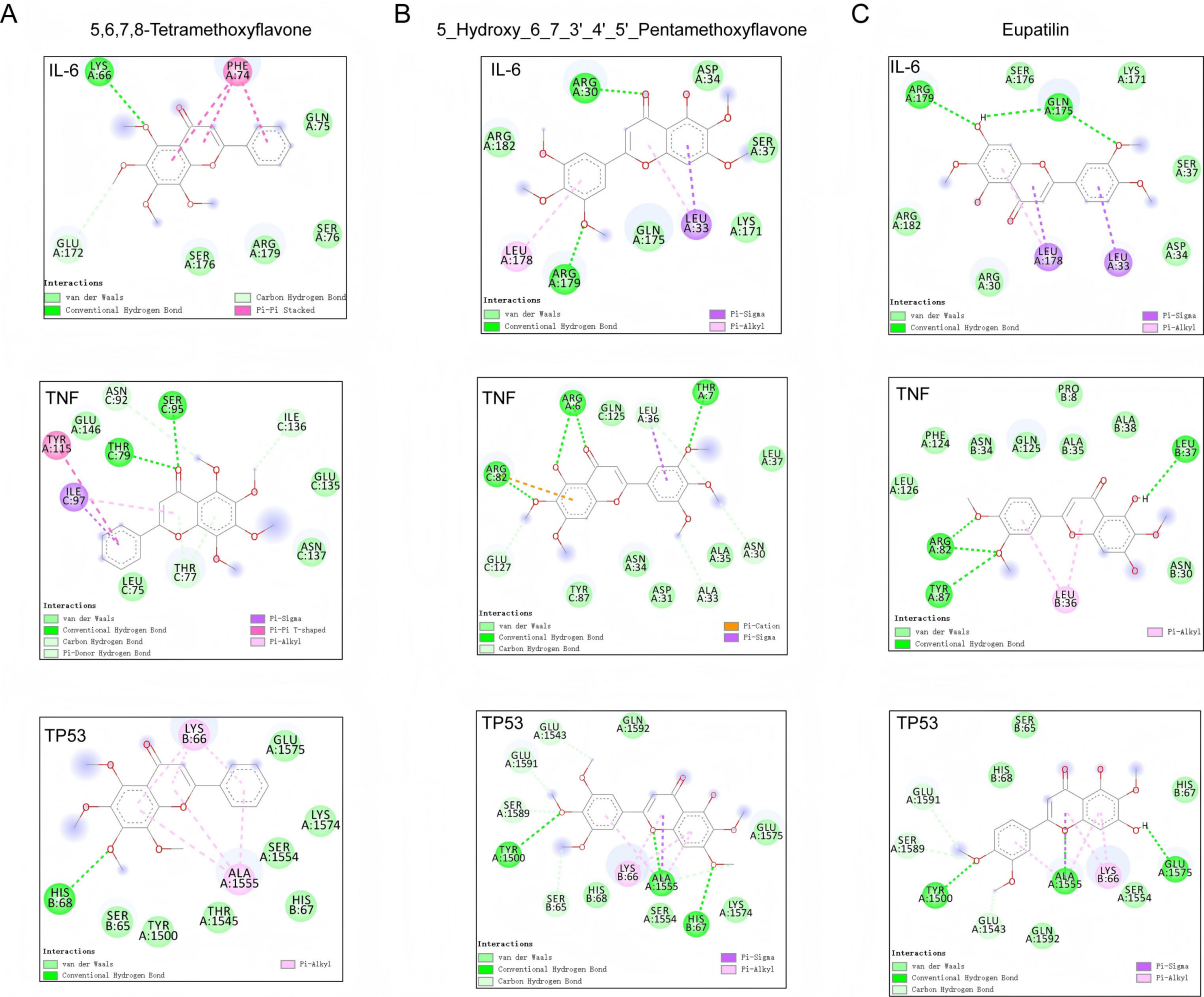
Two‑dimensional (2D) diagrams of the interactions between the top 3 compounds (5,6,7,8‑tetramethoxyflavone **(A)**, 5‑hydroxy‑6,7,3’,4’,5’‑pentamethoxyflavone **(B)**, and eupatilin **(C)**) and top 3 target proteins. Bonds between the compounds and amino acid residues are indicated by colored dashed lines.

Finally, molecular docking analysis was performed on the top 3 bioactive components of Liubao tea and their corresponding core hub targets identified from the network pharmacology analysis. Results demonstrated that these chemical components, namely eupatilin, 5,6,7,8-tetramethoxyflavone, and 5-hydroxy-6,7,3’,4’,5’-pentamethoxyflavone, exhibited potential binding capabilities with the key targets TP53, IL6, and TNF (**Figures 12A–C**). This finding suggests that Liubao tea may exert its therapeutic effects by binding to these core hub targets.

## 4 Discussion

OA is a globally prevalent chronic joint disorder characterized by progressive cartilage degradation, subchondral bone remodeling, and systemic inflammation, for which limited disease-modifying therapies are available to date (1, 3). Traditional Chinese medicine has emerged as a promising source for developing novel OA treatments owing to its holistic regulatory effects and favorable safety profiles (25). In this study, we systematically investigated the therapeutic potential and underlying mechanisms of Liubao tea, a traditional Chinese dark tea, in a destabilization of the DMM-induced mouse OA model. By integrating network pharmacology, gut microbiota analysis, metabolomics, and targeted inhibitor experiments, we demonstrated that Liubao tea ameliorates OA progression by modulating the composition of the gut microbiota and suppressing the pyrimidine metabolism pathway. These findings offer novel insights into the development of natural product-based therapies for OA.

As a time-honored dark tea, Liubao tea is renowned for its unique fermentation process and abundant bioactive compounds. This distinctive fermentation endows Liubao tea with characteristics such as a vibrant reddish-brown hue, rich mellow taste, aged aroma, and pure quality, which are primarily achieved through complex chemical transformations including oxidation, degradation, methylation, and glycosylation (12). Additionally, Liubao tea contains various bioactive components, such as ellagic acid, catechins, polysaccharides, and theaflavins, which exhibit diverse regulatory activities against oxidative stress, metabolic syndrome, organ damage, and microbial imbalances (12). The potential health benefits of Liubao tea have been extensively investigated. Studies have shown that Liubao tea extract can ameliorate diabetes and obesity-related hyperlipidemia by regulating gut microbiota (15, 26). In diabetic models, this extract not only significantly reduced blood glucose levels but also improved gut microbiota composition by increasing the abundance of beneficial bacteria (27). Based on these anti-inflammatory properties of Liubao tea, it (i.e., Liubao tea) may be beneficial for alleviating the progression of OA. In the present study, we first identified the main chemical components of Liubao tea using mass spectrometry. These metabolites were primarily categorized into flavonoids, phenolic acids, quinones, lignans and coumarins, among others. Notably, these components—such as flavonoids and lignans—have been confirmed to alleviate OA progression (28, 29). However, clear evidence confirming whether Liubao tea itself can ameliorate OA is lacking. In this study, using an OA animal model, we found that Liubao tea treatment alleviated OA-related manifestations in micro-CT analysis, mitigated articular cartilage damage, and reduced inflammatory levels. These results suggest that Liubao tea has the potential to be developed as a natural therapeutic agent for OA.

In recent years, the relationship between gut microbiota and OA has emerged as a research hotspot (24). Studies have indicated that gut microbiota dysbiosis may be a key contributing factor to the occurrence and progression of OA (30, 31). By regulating the body’s immune system, metabolic functions, and inflammatory responses, gut microbiota may play a crucial role in the initiation and progression of OA (32, 33). Studies have shown that OA patients exhibit a significant reduction in the diversity and abundance of gut microbiota, while the relative abundance of certain inflammation-associated bacteria, such as Clostridium spp., is increased (31, 34). These findings suggest that therapeutic strategies for OA can be developed through the targeted modulation of gut microbiota (35, 36). Previous studies have demonstrated that Liubao tea exerts beneficial effects on the composition of gut microbiota. Specifically, Liubao tea extract can increase the abundance of beneficial bacteria such as *Bacteroides*, *Akkermansia*, and *Psychrobacter*, while inhibiting the growth of harmful bacteria including *Dubosiella* and *Faecalibaculum (*30). These changes showed significant correlations with serum lipid levels, body weight gain, and the dosage of Liubao tea extract, indicating that Liubao tea extract exerts a regulatory effect on metabolic disorders through the modulation of gut microbiota (30). Furthermore, Liubao tea extract has also been shown to exert beneficial regulatory effects on diabetes-induced gut microbiota dysbiosis, including increasing the *Bacteroidetes/Bacilliota* ratio and upregulating the abundance of short-chain fatty acid (SCFA)-producing bacteria (32). In the present study, through fecal 16S rRNA gene sequencing analysis, we found that Liubao tea increased the α-diversity of gut microbiota and altered its composition in OA mice. Furthermore, fecal microbiota transplantation experiments revealed that transplantation of fecal microbiota from Liubao tea-treated mice significantly ameliorated OA progression. These results suggest that Liubao tea can ameliorate OA by regulating gut microbiota.

In recent years, studies have indicated that metabolic abnormalities are closely associated with the occurrence and progression of OA (37). These abnormalities include lipid metabolism, glucose metabolism, and amino acid metabolism, among others (38–40). Therefore, we analyzed the effect of Liubao tea on serum metabolites in OA mice using untargeted metabolomics. We found that Liubao tea significantly downregulated the pyrimidine metabolism pathway. Previous studies have reported that dysregulation of the pyrimidine metabolism pathway may lead to neurological, hematological, and immune system diseases, and is also associated with malignant tumors (41). A previous study demonstrated that inhibition of dihydroorotate dehydrogenase (DHODH), a key enzyme in *de novo* pyrimidine biosynthesis, can effectively slow the progression of experimental autoimmune arthritis (42). Additionally, DHODH inhibitors, such as tetrahydroindazoles and acrylamide derivatives, have shown promise in preclinical studies by effectively reducing pyrimidine synthesis and exerting anti-arthritic effects (43, 44). However, the relationship between pyrimidine metabolism and OA remains unclear. In the present study, by inhibiting endogenous pyrimidine synthesis, we found that suppressing pyrimidine metabolism significantly ameliorated OA progression. This suggests that Liubao tea may exert its therapeutic effect on OA by inhibiting pyrimidine metabolism, and future studies are warranted to further explore the mechanism by which pyrimidine metabolism contributes to OA pathogenesis.

In the present study, we identified the chemical constituents of Liubao tea using mass spectrometry analysis. Furthermore, through network pharmacology and molecular docking analyses, we found that the key components of Liubao tea, namely eupatilin, 5,6,7,8-tetramethoxyflavone, and 5-hydroxy-6,7,3’,4’,5’-pentamethoxyflavone, exhibit potential interactions with the core targets TP53, IL6, and TNF. This is consistent with the results of our animal experiments, which demonstrated that Liubao tea reduced the serum levels of IL-6 and TNF-α. In fact, it has been confirmed that eupatilin exerts antinociceptive and chondroprotective properties in a rat model of OA by downregulating oxidative damage and catabolic activity in chondrocytes (45). Additionally, TP53, IL6, and TNF have also been well-documented to be involved in the progression of OA (46–48). Our molecular docking assays suggest that the core components of Liubao tea may exert their therapeutic effects by targeting these key molecules. Further *in vivo* and *in vitro* experiments are warranted to elucidate the specific regulatory mechanisms underlying the effects of Liubao tea on these targets.

## 5 Limitations

However, this study has certain limitations. First, the precise molecular interactions between the identified bioactive components and their predicted targets require further experimental validation. Furthermore, the specific bacterial taxa and microbial metabolites responsible for the observed effects remain to be fully elucidated. Finally, the translational potential of these findings from a murine model to human OA patients warrants future clinical investigation.

## 6 Conclusion

In conclusion, this study demonstrates that Liubao tea effectively ameliorates experimental osteoarthritis by mitigating structural joint damage, reducing inflammation, and restoring subchondral bone architecture. The therapeutic benefits are mediated through a novel mechanism involving the remodeling of gut microbiota and subsequent suppression of the host pyrimidine metabolism pathway. These findings position Liubao tea as a promising natural product-derived candidate for OA intervention.

## Data Availability

The raw sequence data from 16S rRNA sequencing have been deposited in the NCBI database under the accession number PRJNA1419793. The metabolomics data have been deposited in the MetaboLights database with the accession number MTBLS13905.

## References

[1] Kloppenburg M Namane M Cicuttini F . Osteoarthritis. Lancet. (2025) 405:71–85. doi: 10.1016/S0140-6736(24)02322-5, PMID: 39755397

[2] Moulin D Sellam J Berenbaum F Guicheux J Boutet M . The role of the immune system in osteoarthritis: mechanisms, challenges and future directions. Nat Rev Rheumatol. (2025) 21:221–36. doi: 10.1038/s41584-025-01223-y, PMID: 40082724

[3] Liu W Guo N Wang J Xu B . Osteoarthritis: mechanisms and therapeutic advances. MedComm. (2020) . 2025. 6:e70290. doi: 10.1002/mco2.70290, PMID: 40757100 PMC12314552

[4] Matthews GL Hunter DJ . Drugs for osteoarthritis. JAMA. (2021) 325:581–2. doi: 10.1001/jama.2020.8395, PMID: 33560319

[5] Rodriguez-Merchan EC . The current role of disease-modifying osteoarthritis drugs. Arch Bone Jt Surg. (2023) 11:11–22. doi: 10.22038/ABJS.2021.56530.2807, PMID: 36793668 PMC9903308

[6] Feng P Lin Z Liang M Zhang X Meng L Duan T . Research progress on traditional Chinese medicine application in osteoarthritis. Tradit Med Res. (2025) 10:43. doi: 10.53388/TMR20240821001

[7] Zhou Q Liu J Xin L Fang Y Hu Y Qi Y . Association between traditional Chinese Medicine and osteoarthritis outcome: A 5-year matched cohort study. Heliyon. (2024) 10:e26289. doi: 10.1016/j.heliyon.2024.e26289, PMID: 38390046 PMC10881435

[8] Zeng L Zhou G Yang W Liu J . Guidelines for the diagnosis and treatment of knee osteoarthritis with integrative medicine based on traditional Chinese medicine. Front Med (Lausanne). (2023) 10:1260943. doi: 10.3389/fmed.2023.1260943, PMID: 37915321 PMC10617515

[9] Zhou G Zhang X Gu Z Zhao J Luo M . Research progress in single-herb chinese medicine and compound medicine for knee osteoarthritis. Comb Chem High Throughput Screen. (2024) 27:2180–6. doi: 10.2174/0113862073264850231116055745, PMID: 38305402 PMC11348453

[10] Li W Yu L Li W Ge G Ma Y Xiao L . Prevention and treatment of inflammatory arthritis with traditional Chinese medicine: Underlying mechanisms based on cell and molecular targets. Ageing Res Rev. (2023) 89:101981. doi: 10.1016/j.arr.2023.101981, PMID: 37302756

[11] Wu Z Xu H Qiao B . Yiqi Xugu HeJi restores cartilage metabolic homeostasis via AKT1-Thr473 activation in osteoarthritis. Phytomedicine. (2025) 148:157228. doi: 10.1016/j.phymed.2025.157228, PMID: 40966781

[12] Feng X Chen M Song H Ma S Ou C Li Z . A systemic review on Liubao tea: A time-honored dark tea with distinctive raw materials, process techniques, chemical profiles, and biological activities. Compr Rev Food Sci Food Saf. (2023) 22:5063–85. doi: 10.1111/1541-4337.13254, PMID: 37850384

[13] Pan Y Long X Yi R Zhao X . Polyphenols in liubao tea can prevent CCl_4_-induced hepatic damage in mice through its antioxidant capacities. Nutrients. (2018) 10. doi: 10.3390/nu10091280, PMID: 30201943 PMC6163653

[14] Zhou S Bao Z Ma S Ou C Hu H Yang Y . A local dark tea - Liubao tea - extract exhibits remarkable performance in oral tissue regeneration, inflammation relief and oral microbiota reconstruction. Food Funct. (2023) 14:7400–12. doi: 10.1039/D3FO02277C, PMID: 37475617

[15] Feng X Qin Y Ma S Ming S Weng Z Xuan Y . Liubao tea extract restrains obesity-related hyperlipidemia via regulation of AMPK/p38/NF-κB pathway and intestinal microbiota. Food Chem. (2025) 464:141910. doi: 10.1016/j.foodchem.2024.141910, PMID: 39522375

[16] Feng X Chen Y Luo L Fang Z Pan Y Lv H . Liubao insect tea polyphenols ameliorate DSS-induced experimental colitis by protecting intestinal barrier and regulating intestinal microbiota. Food Chem. (2025) 467:142156. doi: 10.1016/j.foodchem.2024.142156, PMID: 39632169

[17] Guo S Shi Y Xu A Wang Y Xu P . Liubao tea extract ameliorates ovalbumin-induced allergic asthma by regulating gut microbiota in mice. Food Funct. (2023) 14:10605–16. doi: 10.1039/D3FO03470D, PMID: 37961950

[18] Luo J Wei Z Tan Y Tong Y Yang B Wen M . Liubao tea extract attenuates high-fat diet and streptozotocin-induced type 2 diabetes in mice by remodeling hepatic metabolism and gut microbiota. Nutrients. (2025) 17. doi: 10.3390/nu17162665, PMID: 40871693 PMC12389309

[19] Li B Liu J He C Deng Z Zhou X Peng R . Unveiling the therapeutic potential of berberine in rheumatoid arthritis: A comprehensive study of network pharmacology, metabolomics, and intestinal flora. J Inflammation Res. (2024) 17:10849–69. doi: 10.2147/JIR.S493892, PMID: 39677295 PMC11645930

[20] Fu Q Liu LG Li W Liu J Shang L Li J . Screening of potentially active compounds against rheumatoid arthritis in the Juan-Bi decoction using systems pharmacology and animal experiments. Front Cell Dev Biol. (2024) 12:1396890. doi: 10.3389/fcell.2024.1396890, PMID: 38983788 PMC11231194

[21] Shen L Li W Chen Y Guan D Lu A Xu A . SuHeXiang Wan in the treatment of stroke: Prediction potentially active metabolites using a combination of in silico analysis and experimental viability assessment. J Pharm BioMed Anal. (2025) 270:117313. doi: 10.1016/j.jpba.2025.117313, PMID: 41389492

[22] Xu A Wen ZH Su SX Chen YP Liu WC Guo SQ . Elucidating the synergistic effect of multiple chinese herbal prescriptions in the treatment of post-stroke neurological damage. Front Pharmacol. (2022) 13:784242. doi: 10.3389/fphar.2022.784242, PMID: 35355727 PMC8959705

[23] Xu A Li W Cai J Wen Z Wang K Chen Y . Screening of key functional components of Taohong Siwu Decoction on ischemic stroke treatment based on multiobjective optimization approach and experimental validation. BMC Complement Med Ther. (2023) 23:178. doi: 10.1186/s12906-023-03990-1, PMID: 37264383 PMC10234048

[24] Xi Y Wang Z Wei Y . Gut microbiota and osteoarthritis: from pathogenesis to novel therapeutic opportunities. Am J Chin Med. (2025) 53:43–66. doi: 10.1142/S0192415X2550003X, PMID: 39880660

[25] Wang Z Zhao C Li M Zhang L Diao J Wu Y . Efficacy and safety of external therapies of traditional chinese medicine in patients with knee osteoarthritis: A systematic review and network meta-analysis. Rejuvenation Res. (2025) 28:248–62. doi: 10.1089/rej.2025.0039, PMID: 40511477

[26] Ding Q Zhang B Zheng W Chen X Zhang J Yan R . Liupao tea extract alleviates diabetes mellitus and modulates gut microbiota in rats induced by streptozotocin and high-fat, high-sugar diet. BioMed Pharmacother. (2019) 118:109262. doi: 10.1016/j.biopha.2019.109262, PMID: 31376656

[27] Zhu J Wu M Zhou H Cheng L Wei X Wang Y . Liubao brick tea activates the PI3K-Akt signaling pathway to lower blood glucose, metabolic disorders and insulin resistance via altering the intestinal flora. Food Res Int. (2021) 148:110594. doi: 10.1016/j.foodres.2021.110594, PMID: 34507739

[28] Naselli F Bellavia D Costa V De Luca A Raimondi L Giavaresi G . Osteoarthritis in the elderly population: preclinical evidence of nutrigenomic activities of flavonoids. Nutrients. (2023) 16. doi: 10.3390/nu16010112, PMID: 38201942 PMC10780745

[29] Min L Wu Y Cao G Mi D Chen C . A network pharmacology strategy to investigate the anti-osteoarthritis mechanism of main lignans components of Schisandrae Fructus. Int Immunopharmacol. (2021) 98:107873. doi: 10.1016/j.intimp.2021.107873, PMID: 34182246

[30] Hao C Li H Zhang Y Zhang W Sun C Yang T . Osteoarthritis treatment via the GLP-1-mediated gut-joint axis targets intestinal FXR signaling. Science. (2025) 388:eadt0548. doi: 10.1126/science.adt0548, PMID: 40179178

[31] Chen J Wang A Wang Q . Dysbiosis of the gut microbiome is a risk factor for osteoarthritis in older female adults: a case control study. BMC Bioinf. (2021) 22:299. doi: 10.1186/s12859-021-04199-0, PMID: 34082693 PMC8173911

[32] Longo UG Lalli A Bandini B de Sire R Angeletti S Lustig S . Role of the gut microbiota in osteoarthritis, rheumatoid arthritis, and spondylarthritis: an update on the gut-joint axis. Int J Mol Sci. (2024) 25. doi: 10.3390/ijms25063242, PMID: 38542216 PMC10970477

[33] Yu F Zhu C Wu W . Senile osteoarthritis regulated by the gut microbiota: from mechanisms to treatments. Int J Mol Sci. (2025) 26. doi: 10.3390/ijms26041505, PMID: 40003971 PMC11855920

[34] Liu S Li G Zhu Y Xu C Yang Q Xiong A . Analysis of gut microbiome composition, function, and phenotype in patients with osteoarthritis. Front Microbiol. (2022) 13:980591. doi: 10.3389/fmicb.2022.980591, PMID: 36504782 PMC9732244

[35] Huang M Liu C Huang Y He T Dong Z . Genetic association of gut microbiota with osteoarthritis: a multivariable Mendelian randomization study considering medication use. J Med Microbiol. (2024) 73. doi: 10.1099/jmm.0.001920, PMID: 39412235

[36] Wei Z Li F Pi G . Association between gut microbiota and osteoarthritis: A review of evidence for potential mechanisms and therapeutics. Front Cell Infect Microbiol. (2022) 12:812596. doi: 10.3389/fcimb.2022.812596, PMID: 35372125 PMC8966131

[37] Akhbari P Karamchandani U Jaggard MKJ Graça G Bhattacharya R Lindon JC . Can joint fluid metabolic profiling (or “metabonomics”) reveal biomarkers for osteoarthritis and inflammatory joint disease?: A systematic review. Bone Joint Res. (2020) 9:108–19. doi: 10.1302/2046-3758.93.BJR-2019-0167.R1, PMID: 32435463 PMC7229296

[38] Jiang H Jiang R Wang R Li W Tan G Lv Z . SESN2 maintains cartilage homeostasis by SREBP1-mediated lipid metabolism during osteoarthritis progression. iScience. (2025) 28:113097. doi: 10.1016/j.isci.2025.113097, PMID: 40822351 PMC12356327

[39] Liao Z Cai X Zheng Y Lin J Yang X Lin W . Sirtuin 1 in osteoarthritis: Perspectives on regulating glucose metabolism. Pharmacol Res. (2024) 202:107141. doi: 10.1016/j.phrs.2024.107141, PMID: 38490314

[40] Li Y Xiao W Luo W Zeng C Deng Z Ren W . Alterations of amino acid metabolism in osteoarthritis: its implications for nutrition and health. Amino Acids. (2016) 48:907–14. doi: 10.1007/s00726-015-2168-x, PMID: 26767374

[41] Vincenzetti S Polzonetti V Micozzi D Pucciarelli S . Enzymology of pyrimidine metabolism and neurodegeneration. Curr Med Chem. (2016) 23:1408–31. doi: 10.2174/0929867323666160411125803, PMID: 27063261

[42] Peres RS Santos GB Cecilio NT Jabor VA Niehues M Buqui G . Lapachol, a compound targeting pyrimidine metabolism, ameliorates experimental autoimmune arthritis. Arthritis Res Ther. (2017) 19:47. doi: 10.1186/s13075-017-1236-x, PMID: 28270195 PMC5341405

[43] Zeng F Li S Yang G Qi T Liang Y Yang T . Design, synthesis, molecular modeling, and biological evaluation of acrylamide derivatives as potent inhibitors of human dihydroorotate dehydrogenase for the treatment of rheumatoid arthritis. Acta Pharm Sin B. (2020). doi: 10.1016/j.apsb.2020.10.008, PMID: 33078092 PMC7558257

[44] Popova G Ladds MJGW Johansson L Saleh A Larsson J Sandberg L . Optimization of tetrahydroindazoles as inhibitors of human dihydroorotate dehydrogenase and evaluation of their activity and *in vitro* metabolic stability. J Med Chem. (2020) 63:3915–34. doi: 10.1021/acs.jmedchem.9b01658, PMID: 32212728

[45] Jeong JH Moon SJ Jhun JY Yang EJ Cho ML Min JK . Eupatilin exerts antinociceptive and chondroprotective properties in a rat model of osteoarthritis by downregulating oxidative damage and catabolic activity in chondrocytes. PloS One. (2015) 10:e0130882. doi: 10.1371/journal.pone.0130882, PMID: 26083352 PMC4471346

[46] Zhou Q Liu J Qi Y Hu Y Li Y Cong C . Jianpi qingre tongluo prescription alleviates the senescence-associated secretory phenotype with osteoarthritis by regulating STAG1/TP53/P21 signaling pathway. J Ethnopharmacol. (2025) 337:118953. doi: 10.1016/j.jep.2024.118953, PMID: 39423944

[47] Wiegertjes R van de Loo F Blaney Davidson EN . A roadmap to target interleukin-6 in osteoarthritis. Rheumatol (Oxford). (2020) 59:2681–94. doi: 10.1093/rheumatology/keaa248, PMID: 32691066 PMC7516110

[48] Vlachogiannis NI Evangelou K Ntari L Nikolaou C Denis MC Karagianni N . Targeting senescence and inflammation in chronic destructive TNF-driven joint pathology. Mech Ageing Dev. (2023) 214:111856. doi: 10.1016/j.mad.2023.111856, PMID: 37558168

